# A Bibliometric Analysis of Top-Cited Journal Articles in Obstetrics and Gynecology

**DOI:** 10.1001/jamanetworkopen.2019.18007

**Published:** 2019-12-20

**Authors:** Justin S. Brandt, Ola Hadaya, Meike Schuster, Todd Rosen, Mark V. Sauer, Cande V. Ananth

**Affiliations:** 1 Division of Maternal-Fetal Medicine, Department of Obstetrics, Gynecology and Reproductive Sciences, Rutgers Robert Wood Johnson Medical School, New Brunswick, New Jersey; 2 Department of Obstetrics, Gynecology and Reproductive Sciences, Rutgers Robert Wood Johnson Medical School, New Brunswick, New Jersey; 3 Division of Epidemiology and Biostatistics, Department of Obstetrics, Gynecology and Reproductive Sciences, Rutgers Robert Wood Johnson Medical School, New Brunswick, New Jersey; 4 Department of Biostatistics and Epidemiology, Rutgers School of Public Health, Piscataway, New Jersey; 5 Environmental and Occupational Health Sciences Institute, Rutgers Robert Wood Johnson Medical School, Piscataway, New Jersey

## Abstract

**Importance:**

Citation analysis is a bibliometric method that uses citation rates to evaluate research performance. This type of analysis can identify the articles that have shaped the modern history of obstetrics and gynecology (OBGYN).

**Objectives:**

To identify and characterize top-cited OBGYN articles in the Institute for Scientific Information Web of Science’s Science Citation Index Expanded and to compare top-cited OBGYN articles published in specialty OBGYN journals with those published in nonspecialty journals.

**Design, Setting, and Participants:**

Cross-sectional bibliometric analysis of top-cited articles that were indexed in the Science Citation Index Expanded from 1980 to 2018. The Science Citation Index Expanded was queried using search terms from the American Board of Obstetrics and Gynecology’s 2018 certifying examination topics list. The top 100 articles from all journals and the top 100 articles from OBGYN journals were evaluated for specific characteristics. Data were analyzed in March 2019.

**Main Outcomes and Measures:**

The articles were characterized by citation number, publication year, topic, study design, and authorship. After excluding articles that featured on both lists, top-cited articles were compared.

**Results:**

The query identified 3 767 874 articles, of which 278 846 (7.4%) were published in OBGYN journals. The top-cited article was published by Rossouw and colleagues in *JAMA* (2002). Top-cited articles published in nonspecialty journals were more frequently cited than those in OBGYN journals (median [interquartile range], 1738 [1490-2077] citations vs 666 [580-843] citations, respectively; *P* < .001) and were more likely to be randomized trials (25.0% vs 2.2%, respectively; difference, 22.8%; 95% CI, 13.5%-32.2%; *P* < .001). Whereas articles from nonspecialty journals focused on broad topics like osteoporosis, articles from OBGYN journal focused on topics like preeclampsia and endometriosis.

**Conclusions and Relevance:**

This study found substantial differences between top-cited OBGYN articles published in nonspecialty vs OBGYN journals. These differences may reflect the different goals of the journals, which work together to ensure optimal dissemination of impactful articles.

## Key Points

**Question:**

What are the top-cited obstetrics and gynecology (OBGYN) articles in the Institute for Scientific Information Web of Science’s Science Citation Index Expanded and how do the articles from nonspecialty journals compare with those published in OBGYN specialty journals?

**Findings:**

In this cross-sectional bibliometric analysis, search terms from the American Board of Obstetrics and Gynecology’s 2018 certifying examination topics list were used to identify top-cited articles from 1980 to 2018. Compared with top-cited articles published in OBGYN journals, those published in nonspecialty journals covered topics with broad interest to women’s health care professionals, were more frequently cited, and were more likely to be randomized trials.

**Meaning:**

There are substantial differences between top-cited OBGYN articles published in nonspecialty vs OBGYN journals, which likely reflect the different goals of the journals.

## Teaser

This cross-sectional bibliometric analysis identifies top-cited articles on obstetrics and gynecology topics and compares top-cited articles published in obstetrics and gynecology specialty journals with those published in general medical journals.

## Introduction

Evaluative bibliometrics is a field of quantitative science that uses methods like citation analysis to evaluate research performance.^1^ Citation data are used to quantify the impact of an article over time as indicated by the number of times articles have been cited. Academic success is largely predicated on the productive authorship of highly cited articles,^2,3^ and a bibliometric analysis can serve to identify influential articles that have shaped medical practice and fostered new research ideas.

The term *citation classics* was introduced in 1955 by Eugene Garfield, PhD, to identify top-cited scientific articles in the Institute for Scientific Information (ISI) Web of Knowledge (now known as the Web of Science) databases.^4^ His intent was to capture the “human side of science” and include “personal details that are rarely found in formal academic publication, such as obstacles encountered and byways taken.”^4^ The ISI Web of Science has grown substantially since Dr Garfield’s day. In its current form, the ISI Web of Science indexes scientific publishing databases including Science Citation Index Expanded (SCIE), Current Concepts, and MEDLINE. In most fields, an article that is cited more than 100 times is considered a citation classic, although higher cutoffs (eg, >400 citations) have been used in larger fields.^4^

Dr Garfield’s work in *JAMA* traces back to 1987, with the article “100 Citation Classics From the *Journal of the American Medical Association*.”^5^ Since then, the JAMA Network has been a platform for several bibliometric studies, publishing the citation classics of dermatology, ophthalmology, and critical care.^6,7,8^ However, the citation classics of obstetrics and gynecology (OBGYN) have not yet been profiled in the JAMA Network.

Prior bibliometric studies have identified and characterized frequently cited articles in OBGYN^9^ and human reproduction.^10^ Studies have also examined top-cited articles in high-impact OBGYN journals, such as the *American Journal of Obstetrics and Gynecology*^11^ and *Fertility and Sterility*.^12,13^ Although these studies identified many OBGYN citation classics, they were limited to top-cited articles in OBGYN journals. Nonspecialty general medicine and surgery journals, which generally reach broader audiences and have higher journal impact factors, were excluded from these analyses.

We undertook a bibliometric analysis to identify the top 100 citation classics in OBGYN that were published across all journals. We also compared top-cited OBGYN articles published in OBGYN journals with those published in nonspecialty journals.

## Methods

### Bibliometric Approach

**Figure 1.**
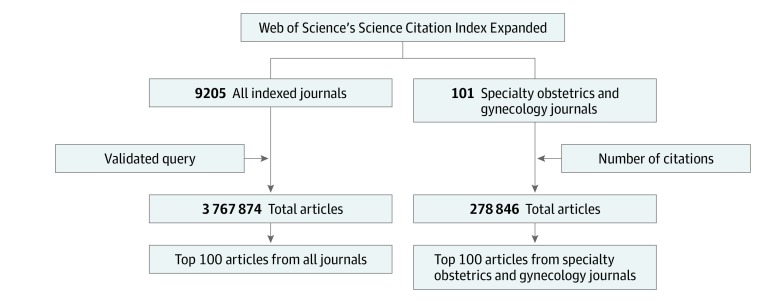
Study Flow Diagram

We used the ISI Web of Science Core Collection^14^ and InCites Journal Citation Reports (JCR)^15^ to identify the most frequently cited OBGYN articles in OBGYN and nonspecialty journals (Figure 1). The ISI Web of Science Core Collection covers articles published from 1980 to 2019 and includes the SCIE as well as other citation indexes. InCites JCR, which categorizes journals by field or specialty, contains citation data from 1997 to 2018. The data used in this study are publicly available and contain no protected health information. Therefore, Rutgers University’s institutional review board approval was not sought. This study followed the Strengthening the Reporting of Observational Studies in Epidemiology (STROBE) reporting guideline for cross-sectional studies.

### Top 100 Articles From All Journals

We queried the SCIE database using search terms from the American Board of Obstetrics and Gynecology (ABOG) 2018 certifying examination topics list^16^ (ABOG confers board certification to diplomates in OBGYN who have passed the written and oral certifying examinations). The certifying examination topics list, which is published annually, outlines the core clinical topics that are deemed essential by ABOG for the independent practice of OBGYN.^16^

To validate the search terms, we restricted the query to OBGYN journals (categorized by SCIE and InCites JCR). We compared the 100 top-cited articles identified by the query with top-cited articles sorted by citation number. We then added search terms (eAppendix 1 in the Supplement) to the query until more than 99% of specialty articles were identified (search schema is shown in eAppendix 2 in the Supplement). After this validation process, we queried the SCIE using the expanded search terms (search schema is shown in eAppendix 3 in the Supplement). We included English-language articles published in all indexed journals from 1980 to 2018 and selected the 100 top-cited OBGYN articles for further review. Articles that were not related to OBGYN were excluded.

### Top 100 Articles From Specialty Journals

We used the ISI Web of Science Core Collection and InCites JCR to identify all indexed specialty OBGYN journals. Under the OBGYN category, we identified 101 journals, including 82 active journals. We queried these journals for English-language articles that were published from 1980 to 2018. These articles were sorted by citation number, and we selected the 100 top-cited articles for further review.

### Study Characteristics

The top 100 articles from all journals and the top 100 articles from OBGYN journals were evaluated for specific characteristics including citation number, publication year, topic, study design (basic and translational science studies, cohort or case-control studies, reviews, meta-analyses, randomized clinical trials [RCTs], new procedure or case reports, and other), journal, and country of origin and institution. Examples of obstetrics topics included preterm labor, preeclampsia, fetal growth restriction, and gestational diabetes. Examples of general gynecology topics included contraception, osteoporosis, and hormone therapy. Examples of reproductive endocrinology and infertility topics included assisted reproduction and endocrinology disorders. Examples of gynecologic oncology topics included the malignant neoplasms of female reproductive organs, such as the cervix, uterus, and ovaries. Examples of urogynecology topics included urinary incontinence and pelvic organ prolapse.

### Statistical Analysis

We compared the top-cited OBGYN articles published in nonspecialty journals with the top-cited articles published in specialty OBGYN journals using the χ^2^ test and Fisher exact probability test (categorical variables), the Wilcoxon rank sum test (nonparametric continuous variables), and the *t* test (parametric continuous variables). Statistical significance was set at 2-tailed *P* < .05. Statistical analyses were performed using Stata version 10.1 (StataCorp LP) and SAS version 9.4 (SAS Institute).

## Results

Articles were queried from the SCIE on November 16, 2018. A total of 3 767 874 articles were identified (Figure 1), of which 278 846 (7.4%) were published in OBGYN journals. In all, 178 200 articles (4.7%) were cited more than 100 times in all indexed journals, 171 815 (4.9%) were cited more than 100 times in nonspecialty journals, and 6385 (2.3%) were cited more than 100 times in OBGYN journals.

### Characteristics of Top 100 Articles From All Journals

The 100 top-cited articles from all journals were published from 1983 to 2011 and can be found in eTable 1 in the Supplement. The median (interquartile range [IQR]) year of article publication was 1999 (1995-2003). The articles received a median (IQR) of 1738 (1464-2097) citations and had a median (IQR) of 99 (74-139) citations per year.

The 100 top-cited OBGYN articles were published in 41 journals (eTable 2 in the Supplement). In all, 25 articles were published in *The New England Journal of Medicine*, 14 articles were published in *Lancet*, and 10 were published in *JAMA*. Only 8 of the top 100 articles were published in OBGYN journals.

The top-cited article was published by Rossouw et al^17^ in *JAMA* in 2002 and received 9204 citations. Although the second most frequently cited article was published by Slamon and colleagues,^18^ the Sub-committee of the International Continence Society’s report on terminology of lower urinary tract function was published simultaneously in 3 journals^19,20,21^ and generated a total of 5883 citations. The authors with the greatest number of top-cited articles are listed in eTable 3 in the Supplement.

Sixty-five articles originated from academic institutions within the United States (eTable 4 in the Supplement). The most common type of article was review (25 articles). There were 23 RCTs and 19 basic science articles. The least common study designs were consensus statement and systematic review and meta-analysis.

The top-cited OBGYN articles covered a range of topics. A total of 37 articles were related to general gynecology topics, with the most prominent being osteoporosis (13 articles). Hormone therapy (8 articles) was a highly cited topic, with articles focusing on the impact on bone health, cardiovascular disease, and gynecologic malignant neoplasms. The other 2 fields with large contributions were obstetrics (30 articles) and gynecologic oncology (24 articles). Frequent topics in obstetrics were preeclampsia (6 articles), fetal programming (4 articles), and gestational diabetes (4 articles). Frequent topics in gynecologic oncology were cervical cancer and human papillomavirus (12 articles) and breast and ovarian cancer and *BRCA* mutations (11 articles).

### Characteristics of Top 100 Articles From OBGYN Journals

The 100 top-cited articles from OBGYN journals were published from 1980 to 2013 (full list in eTable 5 in the Supplement). The median (IQR) year of article publication was 1997 (1989-2004). The articles received a median (IQR) of 671 (590-959) citations and had a median (IQR) of 36 (25-56) citations per year.

The 100 top-cited articles were published in 22 OBGYN journals. Twenty-two articles were published in the *American Journal of Obstetrics and Gynecology*, which makes it the most influential specialty OBGYN journal in this list. In addition, 14 articles were published in *Obstetrics & Gynecology* and 13 articles were published in *Human Reproduction.*

The top-cited article was published by Bump et al^22^ in the *American Journal of Obstetrics and Gynecology* in 1996 and received a total of 2446 citations. However, the “Revised 2003 Consensus on Diagnostic Criteria and Long-term Health Risks Related to Polycystic Ovary Syndrome,” which was published simultaneously in *Fertility and Sterility*^23^ and *Human Reproduction*,^24^ was cited a total of 4303 times, making it the most frequently cited article in specialty OBGYN journals.

Forty-three articles originated from the United States. Thirty-three of the 100 top-cited articles from OBGYN journals were observational studies. There were 24 reviews and 14 basic science articles. There were only 2 RCTs, which was the least common type of study design.

Thirty-one of the 100 top-cited articles were related to obstetrics topics. The most frequent obstetrics topics were preeclampsia (11 articles) and antenatal corticosteroids to promote fetal lung maturity (5 articles). Twenty-two articles were related to reproductive endocrinology and infertility topics, and 20 articles were related to general gynecology topics. The most frequent reproductive endocrinology and infertility topics were infertility and assisted reproduction (13 articles). The most frequent gynecology topic was endometriosis (6 articles).

### Comparison of Top 100 Articles From All Journals vs Top 100 Articles From OBGYN Journals

After excluding the 8 articles on both lists (eTable 6 in the Supplement), we compared top-cited articles published in OBGYN vs nonspecialty journals (Table). The top-cited articles from nonspecialty journals were more frequently cited (median [IQR], 1738 [1490-2077] citations vs 666 [580-843] citations; *P* < .001). More RCTs (25.0% vs 2.2%; difference, 22.8%; 95% CI, 13.5%-32.2%; *P* < .001) and fewer observational studies (13.0% vs 34.8%; difference, −12.5%; 95% CI, −24.9% to 0%; *P* < .001) were published in nonspecialty journals compared with OBGYN journals. In addition, the top-cited articles from nonspecialty journals were more likely to be written by authors from the United States compared with the top-cited articles from OBGYN journals (64.1% vs 46.7%; difference, 17.4%; 95% CI, 3.3%-31.5%; *P* = .02).

**Table.** Analysis Comparing Top-Cited Articles in All Journals With Those Published in OBGYN Journals From 1980 to 2018

Article Characteristic	Journals, No. (%)^a^		*P* Value^b^
	All (n = 92)	Specialty OBGYN (n = 92)	
Year, median (IQR)	1999 (1995-2003)	1997 (1989-2004)	.10
United States	59 (64.1)	43 (46.7)	.02
No. of citations, median (IQR)	1738 (1490-2077)	666 (580-843)	<.001
No. of citations/y, median (IQR)	101 (74-139)	35 (24-51)	<.001
Study type			
Basic or translational	19 (20.7)	14 (15.2)	<.001
Observational	12 (13.0)	32 (34.8)	
Review	23 (25.0)	23 (25.0)	
Consensus	4 (4.4)	7 (7.6)	
Systematic review and meta-analysis	5 (5.4)	8 (8.7)	
Randomized clinical trial	23 (25.0)	2 (2.2)	
New procedure	6 (6.5)	4 (4.4)	
Other	0	2 (2.2)	
Field			
Obstetrics	33 (35.9)	27 (29.4)	<.001
Gynecologic oncology	15 (16.3)	24 (26.1)	
Reproductive endocrinology and Infertility	22 (23.9)	4 (4.4)	
Urogynecology	4 (4.4)	3 (3.3)	
Gynecology	17 (18.5)	34 (37.0)	
Other	1 (1.1)	0	
Open access^c^	30 (32.6)	23 (25.0)	.25

Abbreviations: IQR, interquartile range; OBGYN, obstetrics and gynecology.

^a^ The 8 articles featured on both top-cited lists were excluded from this analysis.

^b^ *P* values were determined by χ^2^ or Fisher exact tests (categorical data) and Wilcoxon rank sum test (for nonparametric continuous data).

^c^ Open access journals are available for free public access.

### Comparing Citation Rates in Specialty OBGYN Journals vs Nonspecialty Journals

**Figure 2.**
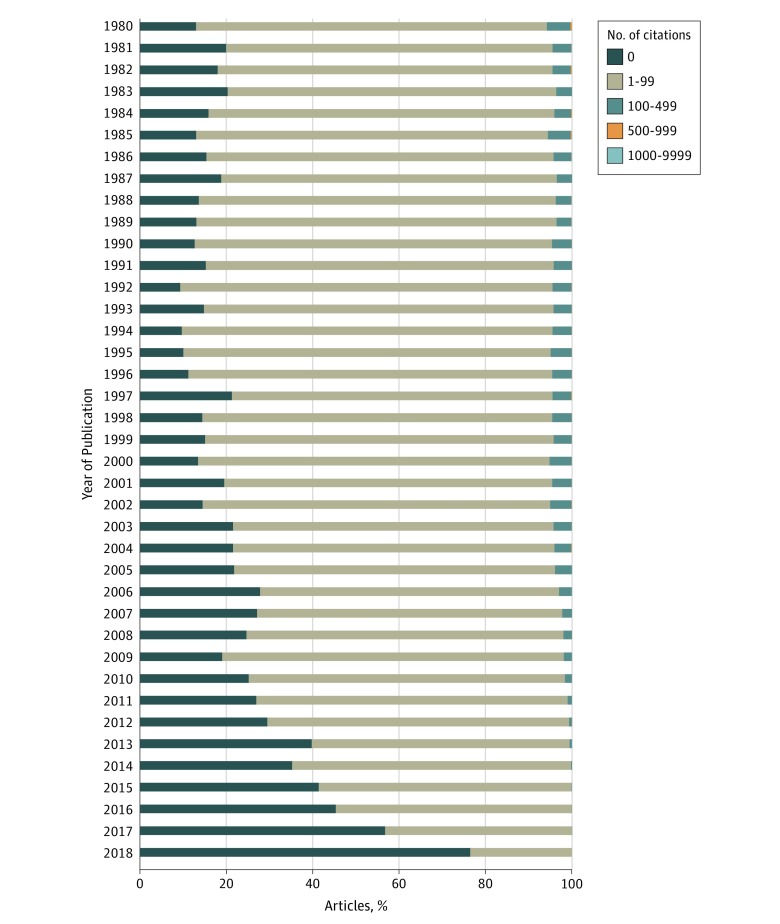
Distribution of Citations by Year of Publication in Obstetrics and Gynecology Journals From 1980 to 2018

**Figure 3.**
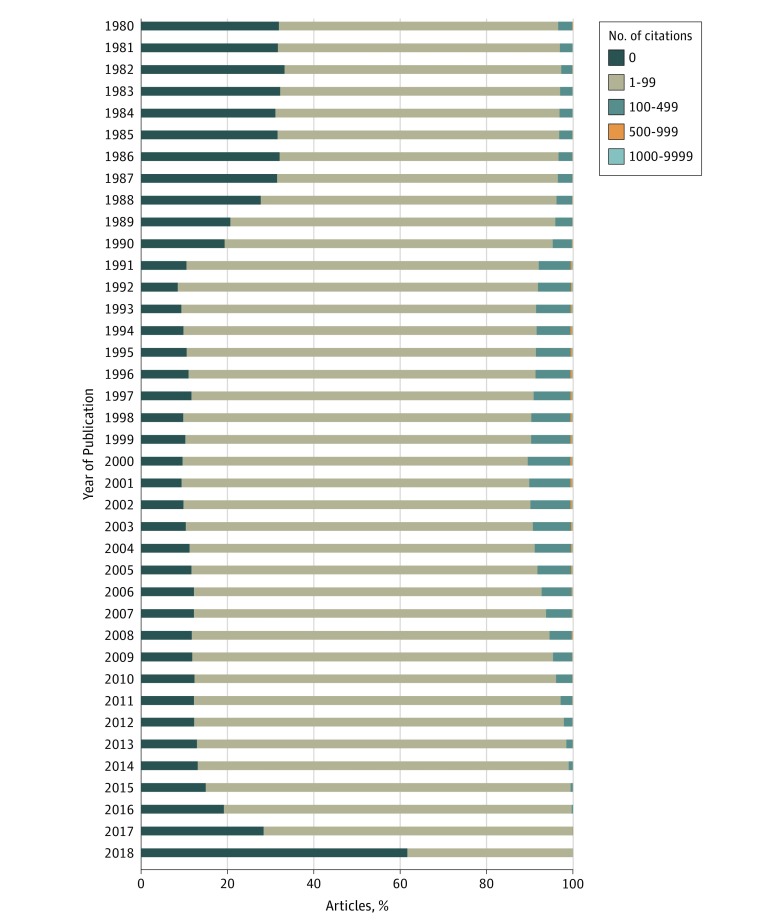
Distribution of Citations by Year of Publication in Nonspecialty Journals From 1980 to 2018

We reviewed temporal trends in citation rates of articles published in OBGYN journals and nonspecialty journals from 1980 to 2018. Nonspecialty journals include all journals indexed in the SCIE that were not categorized as OBGYN journals. Articles published in nonspecialty journals were more likely to have 0 citations (Figure 2 and Figure 3). For articles published in OBGYN journals, 82 257 articles (29.5%) were not cited, 190 204 (68.2%) were cited between 1 and 99 times, and 6267 (2.2%) were cited 100 to 499 times. In contrast, OBGYN articles published in nonspecialty journals had lower rates of 0 citations and higher rates of 1 to 99 and 100 to 499 citations. In nonspecialty journals, 547 669 articles (15.7%) were not cited, 2 769 544 (79.4%) were cited between 1 and 99 times, and 163 098 (4.7%) were cited 100 to 499 times. Less than 1% of articles in both groups were cited more than 500 times. Additional information about the distribution of citations per year of OBGYN articles published in OBGYN and nonspecialty journals is described in eTable 7 through eTable 10 in the Supplement.

The results of our bibliometric study raise important questions about the goals and intentions of OBGYN journals vs nonspecialty journals, which include general medicine and surgery journals. Specialty OBGYN journals must cater to the specialized needs of practitioners within a field. Consequently, the articles published in specialty journals cover more focused topics and are more likely to be observational studies. In contrast, general medicine and surgery journals are not limited by these constraints and can appeal more broadly to readers with greater potential to be highly cited, often publishing articles with higher levels of evidence.

## Discussion

### Principal Findings

We identified the 100 top-cited OBGYN articles that were published in all journals indexed in the SCIE from 1980 and 2018. We identified significant differences between top-cited articles published in nonspecialty vs OBGYN journals. The top-cited OBGYN articles from nonspecialty journals were more frequently cited and had higher levels of evidence. These top-cited articles also covered topics with broad appeal to all women’s health care professionals.

### Results of the Study in Context

We queried the ISI Web of Science using a comprehensive search query based on ABOG’s 2018 certifying examination topics list. Ninety-two of the top-cited OBGYN articles were published in nonspecialty journals. The articles that were published in nonspecialty OBGYN journals have not been previously recognized as citation classics in OBGYN and reproductive medicine.^9,10,11,12,13^ In contrast, a smaller study queried MEDLINE from 2007 to 2013 using major *Medical Subject Headings* of *obstetrics, pregnancy complications,* and *obstetrics surgical procedures*.^25^ That study found that only 10.3% of articles were published in general medicine and surgery journals. Owing to the methods we used here, we performed a more comprehensive assessment of the published literature.

A prior bibliometric study of top-cited specialty OBGYN articles used the Social Sciences Citation Index, which indexed articles from 1956 to 2010.^9^ In that study, top-cited articles were cited in 11 journals, and 49 of the top 100 were published in the *American Journal of Obstetrics and Gynecology*. Our study used the SCIE database, which included articles starting from 1980. As a result, the list of top-cited specialty articles is quite different. In our list, there were 22 unique OBGYN journals that contributed articles to the top 100. Although the *American Journal of Obstetrics and Gynecology* was still responsible for the most articles of any specialty OBGYN journal, the other specialty OBGYN journals made larger contributions than in the previous study.

Of all top-cited OBGYN articles, 25.0% were RCTs, whereas only 2.2% of specialty articles were RCTs. A study of top-cited articles in the *American Journal of Obstetrics and Gynecology* found that significantly more articles after 1995 were RCTs.^11^ Other studies suggest that specialty articles are publishing more studies of higher levels of evidence.^25,26^ Given that we compared top-cited articles from all journals to those published in OBGYN journals across nearly 40 years, our analysis may obscure these relevant, contemporary publication trends.

It remains unclear whether these findings could be observed in other research fields. Interest in bibliometrics has grown substantially since Dr Garfield’s seminal contribution, and today there are many iterations of bibliometric studies.^9,11,27,28,29,30,31,32,33,34,35^ Many have relied on JCR journal categorization to query journals related to a specific field^9^ or combinations of fields.^30^ Studies have looked at individual journals^11^ and groups of journals.^31^ Additionally, studies have used search terms to identify top-cited articles,^28,29,32,33,34,35^ which allows more journals to be queried, although the results are dependent on the search schema. As a consequence of these heterogeneous methods, the various citation analyses have limited comparisons. Further bibliometric methodological research is needed to compare the medical literature across fields.

### Strengths and Limitations

This study has strengths. This bibliometric study is the first of its kind, to our knowledge, to identify and characterize frequently cited OBGYN articles across all journals in the ISI Web of Science SCIE. Most bibliometric studies in OBGYN^9^ and reproductive medicine^10^ have excluded nonspecialty journals. By including all journals in the analysis, we were able to generate a more comprehensive list of top-cited OBGYN articles. To evaluate articles in all journals, we had to develop a unique search strategy. As this may have introduced potential bias as various search strategies, we validated the search query using all indexed specialty OBGYN journals before applying the query to the SCIE.

There are also some weaknesses to the study. Although we used the ISI Web of Science for our bibliometric analysis, there are other public and commercially available bibliometric databases, such as Scopus, Medline, and Google Scholar. No bibliometric database is considered superior, and there are wide variations in citations data in each database.^36,37^ The decision to use the ISI Web of Science SCIE was based on the fact that we wanted to identify the OBGYN citations classics of Dr Garfield.^38^

Because this study was limited to articles that were published after 1980, we missed articles published prior to 1980 that were more frequently cited than those included in this study. For example, “Comparative Aspects of the Brain Growth Spurt” was published in 1979 by Dobbing and Sands^39^ in *Early Human Development*. This article, which was previously recognized as a top-cited article in OBGYN,^9^ was cited 2291 times in MEDLINE (accessed May 10, 2019) but was not identified in our query.

The biggest limitations of our study are inherent to citation analysis, which is based on the absolute number of citations that an article receives. Citation number is a surrogate for influence, but there are many factors that affect citation rates.^3^ For example, this strategy may favor older articles. Journal and author self-citations, incomplete citing, and omission bias also significantly contribute to citation rates.^9,40,41^ In addition, some influential articles are cited a limited number of times until their findings become well known. This bibliometric phenomenon, termed “obliteration by incorporation,”^42^ has been noted in other bibliometric studies.^5,9^

## Conclusions

In this bibliometric study, we queried the SCIE using search terms from the American Board of Obstetrics and Gynecology 2018 certifying examination topics list and identified the top 100 articles from all journals and the top 100 articles from OBGYN journals. The citation classics of several specialties have been profiled in JAMA Network journals,^5,6,7,8^ but only now have we recognized the citation classics of OBYGN.

This study provides insight into how specialty and nonspecialty journals work together to facilitate the dissemination of scientific knowledge in OBGYN. Although their goals are different, these journals work together in a complementary fashion to ensure optimal dissemination of impactful articles to all women’s health care professionals.

## Notes

Supplement. **eAppendix 1.** Search Terms Adapted From the American Board of Obstetric and Gynecology’s 2018 Certifying Examination Topics List **eAppendix 2.** Search Query by Topics in the ISI Web of Knowledge **eAppendix 3.** Search Query by OBGYN Journals in the ISI Web of Knowledge **eTable 1.** 100 Top-Cited Articles From All Journals **eTable 2.** Frequent Journals of 100 Top-Cited Articles From All Journals (Listed if ≥2 Articles Were in Top-Cited List) **eTable 3.** Frequent Authors of 100 Top-Cited Articles From All Journals (Based on 1st, 2nd, and Senior Authorship and Included if ≥2 Papers in Top-Cited List) **eTable 4.** Institutions of Origin of 100 Top-Cited Articles From All Journals (Listed if ≥2 Articles Were in Top-Cited List) **eTable 5.** 100 Top-Cited Articles From OBGYN Journals **eTable 6.** Top-Cited Articles on Both Lists **eTable 7.** Year-by-Year Distribution of the Number of Articles Published in Non-specialty Journals and Cited in the ISI Web of Knowledge’s Science Citation Index Expanded From 1980-2018 **eTable 8.** Year-by-Year Distribution of the Number of Articles Published in OBGYN Journals and Cited in the ISI Web of Knowledge’s Science Citation Index Expanded From 1980-2018 **eTable 9.** Citations by Publication Year for Articles Published in Non-specialty Journals From 1980-2018 **eTable 10.** Citations by Publication Year for Articles Published in OBGYN Journals From 1980-2018
