# Supplementary material for: A Bibliometric Analysis of Top-Cited Journal Articles in Obstetrics and Gynecology
Source: JAMA Netw Open. 2019 Dec 20;2(12):e1918007. doi: 10.1001/jamanetworkopen.2019.18007 (PMC6991228; doi:10.1001/jamanetworkopen.2019.18007)
Supplement: Supplement. — eAppendix 1. Search Terms Adapted From the American Board of Obstetric and Gynecology’s 2018 Certifying Examination Topics List eAppendix 2. Search Query by Topics in the ISI Web of Knowledge eAppendix 3. Search Query by OBGYN Journals in the ISI Web of Knowledge eTable 1. 100 Top-Cited Articles From All Journals eTable 2. Frequent Journals of 100 Top-Cited Articles From All Journals (Listed if ≥2 Articles Were in Top-Cited List) eTable 3. Frequent Authors of 100 Top-Cited Articles From All Journals (Based on 1st, 2nd, and Senior Authorship and Included if ≥2 Papers in Top-Cited List) eTable 4. Institutions of Origin of 100 Top-Cited Articles From All Journals (Listed if ≥2 Articles Were in Top-Cited List) eTable 5. 100 Top-Cited Articles From OBGYN Journals eTable 6. Top-Cited Articles on Both Lists eTable 7. Year-by-Year Distribution of the Number of Articles Published in Non-specialty Journals and Cited in the ISI Web of Knowledge’s Science Citation Index Expanded From 1980-2018 eTable 8. Year-by-Year Distribution of the Number of Articles Published in OBGYN Journals and Cited in the ISI Web of Knowledge’s Science Citation Index Expanded From 1980-2018 eTable 9. Citations by Publication Year for Articles Published in Non-specialty Journals From 1980-2018 eTable 10. Citations by Publication Year for Articles Published in OBGYN Journals From 1980-2018 [file jamanetwopen-2-e1918007-s001.pdf]

Supplementary Online Content

Brandt JS, Hadaya O, Schuster M, Rosen T, Sauer MV, Ananth CV. A bibliometric analysis of top-cited journal articles in obstetrics and gynecology. *JAMA Netw Open*. 2019;2(12):e1918007. doi:10.1001/jamanetworkopen.2019.18007

eAppendix 1. Search Terms Adapted From the American Board of Obstetric and Gynecology's 2018 Certifying Examination Topics List
eAppendix 2. Search Query by Topics in the ISI Web of Knowledge
eAppendix 3. Search Query by OBGYN Journals in the ISI Web of Knowledge
eTable 1. 100 Top-Cited Articles From All Journals
eTable 2. Frequent Journals of 100 Top-Cited Articles From All Journals (Listed if ≥2 Articles Were in Top-Cited List)
eTable 3. Frequent Authors of 100 Top-Cited Articles From All Journals (Based on 1st, 2nd, and Senior Authorship and Included if ≥2 Papers in Top-Cited List)
eTable 4. Institutions of Origin of 100 Top-Cited Articles From All Journals (Listed if ≥2 Articles Were in Top-Cited List)
eTable 5. 100 Top-Cited Articles From OBGYN Journals
eTable 6. Top-Cited Articles on Both Lists
eTable 7. Year-by-Year Distribution of the Number of Articles Published in Non-specialty Journals and Cited in the ISI Web of Knowledge's Science Citation Index Expanded From 1980-2018
eTable 8. Year-by-Year Distribution of the Number of Articles Published in OBGYN Journals and Cited in the ISI Web of Knowledge's Science Citation Index Expanded From 1980-2018
eTable 9. Citations by Publication Year for Articles Published in Non-specialty Journals From 1980-2018
eTable 10. Citations by Publication Year for Articles Published in OBGYN Journals From 1980-2018

This supplementary material has been provided by the authors to give readers additional information about their work.

© 2019 Brandt JS et al. *JAMA Network Open.*

eAppendix 1:

Search terms adapted from the American Board of Obstetric and Gynecology's

2018 *Certifying Examination Topics List*

1. Gynecology
2. Female sexual dysfunction
3. Anorgasmia
4. Anhedonia
5. Dyspareunia
6. Vulvodynia
7. Contraception
8. Family planning
9. Elective termination
10. ETOP
11. Termination of pregnancy
12. IUD
13. Intrauterine device
14. Levonorgestrel implant
15. Levonorgestrel implant IUD
16. Mirena
17. Nexplanon
18. Implanon
19. Oral contraceptive pills
20. OCP
21. BCP
22. Birth control pills
23. Hormonal birth control
24. Birth control
25. Short acting contraception
26. Long acting reversible contraception
27. LARC
28. LARCs
29. Estrogen patch
30. Etonogestrel implant
31. Subdermal implant
32. Infertility
33. Primary infertility
34. Secondary infertility
35. Infertility evaluation
36. Evaluation of infertility
37. Infertility management
38. Management of infertility
39. Endometriosis
40. Diagnosis of endometriosis
41. Evaluation of endometriosis
42. Surgery for endometriosis
43. Menopause
44. Perimenopause
45. Menopausal symptoms
46. Post menopause
47. Postmenopause
48. Postmenopausal
49. Genitourinary syndrome of menopause
50. Hormone replacement therapy
51. Hormone therapy
52. Estrogen therapy
53. Pediatric gynecology
54. Adolescent gynecology
55. Pediatric and adolescent gynecology
56. Congenital abnormalities of female reproductive tract
57. Mullerian
58. PCOS
59. Polycystic ovarian syndrome
60. Polycystic ovaries
61. Polycystic ovary
62. Hirsutism
63. Pelvic pain
64. Chronic pelvic pain
65. Vagina
66. Diseases of the vagina
67. Vaginal disease
68. Vaginitis
69. Vaginal infections
70. Vaginal dysplasia
71. VAIN
72. Vaginal intraepithelial neoplasia
73. Vulva
74. Vaginal dermatitis
75. vulvar dermatitis
76. Vulvar sclerosis
77. Vulvar lichen
78. Vulvar dysplasia
79. Vulvar intraepithelial neoplasia
80. VIN
81. Incontinence
82. Urinary incontinence
83. Mixed incontinence
84. Stress incontinence
85. Urge incontinence

© 2019 Brandt JS et al. *JAMA Network Open.*

86. Overactive bladder
87. OAB
88. Sexually transmitted disease
89. Sexually transmitted infection
90. STD
91. STI
92. Myoma
93. Fibroid
94. Leiomyomata
95. Uterine myomata
96. Myomata
97. Leiomyoma
98. GYN
99. Gynecologic office surgery
100. Office surgery in gynecology
101. GYN office surgery
102. Office surgery in GYN
103. Gynecologic biopsy
104. Hysteroscopy
105. LEEP
106. Loop electrosurgical excision procedure
107. Endometrial biopsy
108. EMB
109. Cervical cytology
110. Abnormal cervical cytology
111. Cervical dysplasia
112. LSIL
113. LGSIL
114. Low grade squamous intraepithelial lesion
115. HSIL
116. HGSIL
117. High grade squamous intraepithelial lesion
118. Cervical intraepithelial neoplasia
119. CIN
120. CIN 1
121. CIN2
122. CIN 3
123. Cervical cancer
124. Cervix cancer
125. Cancer of cervix
126. Carcinoma in situ of cervix
127. Cervical carcinoma in situ
128. Invasive cervical cancer
129. Invasive cervical carcinoma
130. Colposcopy
131. Cervical biopsy
132. Gynecologic ultrasonography
133. Pelvic ultrasound
134. Hysterosalpingogram
135. Hysterosalpingography
136. HSG
137. Sonohysterograms
138. Gynecologic ultrasound
139. Adnexal masses
140. Pelvic masses
141. Benign pelvic masses
142. Benign adnexal masses
143. Pelvic tumors
144. Adnexal tumors
145. Pelvic floor defects
146. Anterior wall prolapse
147. Posterior wall prolapse
148. Enterocele
149. Rectocele
150. Uterine prolapse
151. Cystocele
152. Procidentia
153. Pelvic organ
154. Pelvic organ prolapse
155. Gynecologic surgery
156. Hysterectomy
157. Abdominal hysterectomy
158. Vaginal hysterectomy
159. Robotic hysterectomy
160. Myomectomy
161. Abdominal Myomectomy
162. Vaginal Myomectomy
163. Robotic Myomectomy
164. Gynecologic laparoscopy
165. Diagnostic gynecologic laparoscopy
166. Operative gynecologic laparoscopy
167. Diagnostic laparoscopy in gynecology
168. Operative laparoscopy in gynecology
169. Laparoscopy in gynecology
170. Gynecologic laparotomy
171. Laparotomy in gynecology
172. Operative hysteroscopy
173. Ovary
174. ovarian
175. Uterus
176. Uterine
177. Intrauterine
178. Fallopian tubes
179. Uterine tube
180. Disorders of menstruation
181. Menstrual irregularities
182. Irregularities of menstruation
183. Abnormal uterine bleeding
184. Dysfunctional uterine bleeding
185. Amenorrhea
186. Dysmenorrhea
187. Menorrhagia
188. Menometrorrhagia
189. Intermenstrual bleeding

© 2019 Brandt JS et al. *JAMA Network Open.*

190. AUB
191. DUB
192. PALM-COEIN
193. Postmenopausal bleeding
194. Adenomyosis
195. Surgery for adenomyosis
196. Ectopic pregnancy
197. Tubal pregnancy
198. Pregnancy unknown location
199. PUL
200. Pelvic inflammatory disease
201. PID
202. Cervicitis
203. Tuboovarian abscess
204. TOA
205. Rectovaginal fistula
206. Urinary tract fistula
207. Gynecologic fistula
208. Fistula
209. Gestational trophoblastic disease
210. Gestational trophoblastic neoplasia
211. GTN
212. GTD
213. Molar pregnancy
214. Hydatidiform mole
215. Partial mole
216. Complete mole
217. Abortion
218. Miscarriage
219. Spontaneous abortion
220. Incomplete abortion
221. Complete abortion
222. Septic abortion
223. Spontaneous miscarriage
224. Incomplete miscarriage
225. Complete miscarriage
226. Septic miscarriage
227. SAB
228. Medical management of abortion
229. Medical management of first trimester loss
230. Surgical management of abortion
231. Surgical management of second trimester loss
232. Inevitable abortion
233. First trimester loss
234. First trimester miscarriage
235. First trimester abortion
236. Early miscarriage
237. Early abortion
238. Threatened abortion
239. Threatened miscarriage
240. Dilation and curettage
241. D&C
242. Dilation and evacuation
243. D&E
244. OB and GYN
245. Obstetric
246. Obstetrics and gynecology
247. Preconception
248. Preconception care
249. Preconception counseling
250. Preconception genetic counseling
251. obstetrics and gynecology
252. Teratogens
253. Teratogenesis
254. Organogenesis
255. Congenital malformations
256. Birth defect
257. Congenital anomaly
258. Hyperemesis
259. Nausea and vomiting of pregnancy
260. Hyperemesis gravidarum
261. HEG
262. 2nd trimester loss
263. 3rd trimester loss
264. Second trimester loss
265. Third trimester loss
266. 2nd trimester abortion
267. Second trimester abortion
268. Intrauterine fetal demise
269. 2nd tri abortion
270. Second tri abortion
271. Stillbirth
272. Fetal demise
273. Fetal loss
274. Fetus
275. Fetal
276. Fetal malformation
277. Neonate
278. Fetal development
279. Development of fetus
280. Multifetal gestation
281. Multifetal pregnancy
282. Multiple gestation
283. Multiple pregnancy
284. Twin gestation
285. Higher order multiple
286. Amniocentesis
287. Invasive genetic testing in pregnancy
288. Prenatal diagnosis
289. Chorionic villus sampling
290. CVS
291. Cell free DNA
292. Cell free fetal DNA
293. NIPT

© 2019 Brandt JS et al. *JAMA Network Open.*

294. NIPS
295. First trimester genetic screening
296. First trimester genetic testing
297. First trimester screen
298. Aneuploidy screening
299. Second trimester screen
300. Quad screen
301. Quadruple screen
302. Triple screen
303. Maternal serum analyte
304. Aneuploidy screening
305. Down syndrome screening
306. Obstetric ultrasound
307. Ultrasound in pregnancy
308. Ultrasound in obstetrics
309. Obstetric ultrasonography
310. Ultrasonography in obstetrics
311. Preterm labor
312. PTL
313. Premature rupture of membranes
314. PROM
315. Premature rupture of membranes at term
316. Term premature rupture of membranes
317. Term PROM)
318. Preterm premature rupture of membranes
319. PPROM
320. Preterm PROM
321. Cerclage
322. History indicated cerclage
323. Ultrasound indicated cerclage
324. Emergency cerclage
325. Rescue cerclage
326. Cervical insufficiency
327. Cervical incompetence
328. Short cervix
329. Cervical shortening
330. Preterm delivery
331. PTD
332. Previable delivery
333. Preterm birth
334. Late preterm delivery
335. Post term pregnancy
336. Post dates pregnancy
337. Post term delivery
338. Post-term
339. Post-dates
340. Postterm
341. Postdates
342. Induction of labor
343. IOL
344. Labor induction
345. Augmentation of labor
346. Labor augmentation
347. Labor abnormalities
348. Abnormalities of labor
349. Labor dystocia
350. Arrest of dilation
351. Arrest of descent
352. Protraction of labor
353. Protracted second stage of labor
354. Protracted first stage of labor
355. Protracted labor
356. Failed induction
357. Cephalopelvic disproportion
358. CPD
359. First stage arrest
360. Second stage arrest
361. Fetal heart rate abnormalities
362. Nonreassuring fetal heart tracing
363. Non-reassuring fetal heart tracing
364. NRFT
365. Fetal decelerations
366. Variable decelerations
367. Late decelerations
368. Minimal variability
369. Absent variability
370. Category one tracing
371. Category two tracing
372. Category three tracing
373. Poor variability
374. Fetal tachycardia
375. Fetal bradycardia
376. Fetal monitoring
377. Electronic fetal monitoring
378. Continuous fetal monitoring
379. Breech presentation
380. Face presentation
381. Malpresentation in labor
382. Malpresentation in pregnancy
383. Fetal malpresentation
384. Malpresentation
385. Prolapsed cord
386. Cord prolapse
387. Umbilical cord entanglement
388. Umbilical cord
389. Operative vaginal deliveries
390. Vacuum assisted vaginal delivery
391. Forceps assisted vaginal delivery
392. Obstetrical forceps
393. Assisted second stage
394. Obstetrical hemorrhage
395. Antepartum hemorrhage
396. Intrapartum hemorrhage
397. Postpartum hemorrhage

© 2019 Brandt JS et al. *JAMA Network Open.*

398. Management of postpartum hemorrhage
399. Maternal
400. Maternal morbidity
401. Vaginal hematoma
402. Perineal hematoma
403. Maternal mortality
404. Primary cesarean delivery
405. Primary cesarean section
406. Cesarean delivery
407. Cesarean section
408. Repeat cesarean delivery
409. Repeat cesarean section
410. Complications of cesarean delivery
411. Complications of cesarean section
412. Postoperative complications of cesarean delivery
413. Postoperative complications of cesarean section
414. Wound infection after cesarean delivery
415. Wound infection after cesarean section
416. Hematoma after cesarean delivery
417. Hematoma after cesarean section
418. Hemorrhage after cesarean section
419. Hemorrhage after cesarean delivery
420. Puerperal infection
421. Post cesarean endometritis
422. Endometritis
423. Post vaginal endometritis
424. Post partum infection
425. Postpartum infection
426. Pelvic thrombophlebitis
427. Septic pelvic thrombophlebitis
428. Obstetric anesthesia
429. Obstetric anesthesia
430. OB anesthesia
431. Complications of OB anesthesia
432. Complications of anesthesia in obstetrics
433. Post epidural hypotension
434. Hypotension after epidural
435. Fetal heart rate abnormalities after epidural
436. Fetal heart tracing after epidural
437. Intrapartum infection
438. Intra-amniotic infection
439. Amnionitis
440. Chorioamnionitis
441. Chorio
442. Hypertensive disorders of pregnancy
443. Hypertension in pregnancy
444. Preeclampsia
445. PEC
446. Pregnancy induced hypertension
447. Gestational hypertension
448. Chronic hypertension in pregnancy
449. CHTN
450. Chronic hypertension with superimposed preeclampsia
451. Eclampsia
452. Toxemia
453. Toxemia of pregnancy
454. Pre-eclampsia
455. Cardiovascular disease in pregnancy
456. Pulmonary disease in pregnancy
457. Renal disease in pregnancy
458. Neurological disease in pregnancy
459. Hematologic disease in pregnancy
460. Endocrine disease in pregnancy
461. Autoimmune disorders of pregnancy
462. Cardiovascular disease complicating pregnancy
463. Pulmonary disease complicating pregnancy
464. Renal disease complicating pregnancy
465. Neurological disease complicating pregnancy
466. Hematologic disease complicating pregnancy
467. Endocrine disease complicating pregnancy
468. Autoimmune disorders of pregnancy
469. Autoimmune disorders complicating pregnancy
470. Infectious diseases in pregnancy
471. Congenital infection
472. Vertical transmission
473. Zika virus
474. Zika infection in pregnancy
475. Zika
476. Zika in pregnancy
477. Group A streptococcus in pregnancy
478. Varicella in pregnancy
479. Pyelonephritis in pregnancy
480. CMV in pregnancy
481. Cytomegalovirus
482. Toxoplasmosis in pregnancy
483. Parvovirus in pregnancy
484. Rubella in pregnancy
485. Herpes in pregnancy
486. TORCH infection in pregnancy
487. Pregnancies complicated by human immunodeficiency virus infection
488. HIV in pregnancy
489. HIV infection in pregnancy

© 2019 Brandt JS et al. *JAMA Network Open.*

490. Human immunodeficiency virus infection in pregnancy
491. Human immunodeficiency virus in pregnancy
492. Pregnancy complicated by HIV
493. Pregnancy complicated by HIV infection
494. Pregnancy complicated by human immunodeficiency virus
495. Pregnancy complicated by human immunodeficiency virus infection
496. Pregnancy complicated by AIDS
497. Acquired immunodeficiency syndrome in pregnancy
498. Pregnancy complicated by acquired immunodeficiency syndrome
499. Postpartum psychosis
500. Post partum psychosis
501. Post-partum psychosis
502. Post-partum depression
503. Post partum depression
504. Psychiatric disease in pregnancy
505. Baby blues
506. Edinburgh
507. Postpartum blues
508. Post-partum blues
509. Post partum blues
510. Postpartum depression
511. Abnormal fetal growth
512. IUGR
513. Intrauterine growth restriction
514. Intrauterine growth retardation
515. Fetal growth restriction
516. FGR
517. Macrosomia
518. Accelerated fetal growth
519. Restricted fetal growth
520. Retarded fetal growth
521. Fetal anomalies
522. Fetal abnormalities
523. Fetal malformation
524. Congenital fetal anomalies
525. Congenital fetal abnormalities
526. Congenital fetal malformations
527. Placental abnormalities
528. Abnormalities of placenta
529. Placenta
530. Trauma in pregnancy
531. Diabetes in pregnancy
532. Gestational diabetes
533. Gestational diabetes mellitus
534. Diabetes mellitus in pregnancy
535. Shoulder dystocia
536. Management of shoulder dystocia
537. Early term delivery
538. Isoimmunization
539. Maternal alloimmunization
540. Maternal isoimmunization
541. Rh isoimmunization
542. Rh-isoimmunization
543. Rh sensitization
544. Hemolytic disease of the newborn
545. Hemolytic disease of the fetus "AND" newborn
546. Rh incompatibility
547. Alloimmunization
548. Isoimmunization in pregnancy
549. Alloimmunization in pregnancy
550. HDFN
551. Uterine hyperplasia
552. Hyperplasia of uterus
553. Endometrial hyperplasia
554. Hyperplasia of endometrium
555. uterine malignancy
556. Uterine cancer
557. Endometrial cancer
558. Endometrial malignancy
559. Ovarian cancer
560. Ovarian malignancy
561. Cancer of the ovary
562. Endometrial carcinoma
563. Uterine carcinoma
564. Ovarian carcinoma
565. Gynecologic malignancy
566. Gynecologic cancer
567. Gynecological cancer
568. Cancer in gynecology
569. Urinary incontinence
570. Urinary continence
571. Parturition
572. Menopausal
573. Pelvic Floor
574. Childbirth
575. Child birth
576. Menses
577. Menstrual
578. Down Syndrome
579. Downs Syndrome
580. Downs-Syndrome
581. Down-Syndrome
582. FIGO
583. FIGO staging
584. International Federation of Gynecology and Obstetrics
585. Trisomy
586. Ovarian failure
587. IVF

© 2019 Brandt JS et al. *JAMA Network Open.*

588. In-vitro fertilization
589. In vitro fertilization
590. Invitro fertilization
591. Fertilization
592. In vitro
593. In-vitro
594. In-vitro-fertilization
595. Embryo
596. Fertility
597. Assisted reproductive technology
598. Urogynecology
599. Urogyn
600. Progesterone
601. Estrogen
602. Ultrasonic
603. Ovarian neoplasm
604. Uterine neoplasm
605. Gynecologic neoplasm

© 2019 Brandt JS et al. *JAMA Network Open.*

eAppendix 2:

Search query by topics in the ISI Web of Knowledge

TS=(Gynecology OR Female sexual dysfunction OR Anorgasmia OR Anhedonia OR Dyspareunia OR Vulvodynia OR Contraception OR Family planning OR Elective termination OR ETOP OR Termination pregnancy OR IUD OR Intrauterine device OR Levonorgestrel implant OR Levonorgestrel implant IUD OR Mirena OR Nexplanon OR Implanon OR Combined contraceptive pills OR CCP OR BCP OR Birth control pills OR Hormonal birth control OR Birth control OR Short acting contraception OR Long acting reversible contraception OR LARC OR LARCs OR Estrogen patch OR Etonogestrel implant OR subdermal implant OR Infertility OR Primary infertility OR Secondary infertility OR Infertility evaluation OR Evaluation infertility OR Infertility management OR Management infertility OR Endometriosis OR Diagnosis endometriosis OR Evaluation endometriosis OR Surgery endometriosis OR Menopause OR Perimenopause OR Menopausal symptoms OR Post menopause OR Postmenopause OR Postmenopausal OR Genitourinary syndrome menopause OR Hormone replacement therapy OR Hormone therapy OR Estrogen therapy OR Pediatric gynecology OR Adolescent gynecology OR Pediatric "AND" adolescent gynecology OR Congenital abnormalities female reproductive tract OR Mullerian OR PCOS OR Polycystic ovarian syndrome OR Polycystic ovaries OR Polycystic ovary OR Hirsutism OR Pelvic pain OR Chronic pelvic pain OR Vagina OR Diseases vagina OR Vaginal disease OR Vaginitis OR Vaginal infections OR Vaginal dysplasia OR VAIN OR Vaginal intraepithelial neoplasia OR Vulva OR Vaginal dermatitis OR vulvar dermatitis OR Vulvar sclerosis OR Vulvar lichen OR Vulvar dysplasia OR Vulvar intraepithelial neoplasia OR VIN OR Incontinence OR Urinary incontinence OR Mixed incontinence OR Stress incontinence OR Urge incontinence OR Overactive bladder OR OAB OR Sexually transmitted disease OR Sexually transmitted infection OR STD OR STI OR Myoma OR Fibroid OR Leiomyomata OR Uterine myomata OR Myomata OR Leiomyoma OR GYN OR Gynecologic office surgery OR Office surgery in gynecology OR GYN office surgery OR Office surgery in GYN OR Gynecologic biopsy OR Hysteroscopy OR LEEP OR Loop electrosurgical excision procedure OR Endometrial biopsy OR EMB OR Cervical cytology OR Abnormal cervical cytology OR Cervical dysplasia OR LSIL OR LGSIL OR Low grade squamous intraepithelial lesion OR HSIL OR HGSIL OR High grade squamous intraepithelial lesion OR Cervical intraepithelial neoplasia OR CIN OR CIN 1 OR CIN2 OR CIN 3 OR ovarian neoplasm OR uterine neooplasm OR gynecologic neoplasm OR Cervical cancer OR Cervix cancer OR Cancer cervix OR Carcinoma in situ cervix OR Cervical carcinoma in situ OR Invasive cervical cancer OR Invasive cervical carcinoma OR Colposcopy OR Cervical biopsy OR Gynecologic ultrasonography OR Pelvic ultrasound OR Ultrasonic OR Hysterosalpingogram OR Hysterosalpingography OR HSG OR sonohysterogram OR gynecologic ultrasound OR Adnexal masses OR Pelvic masses OR Benign pelvic masses OR Benign adnexal masses OR Pelvic tumors OR Adnexal tumors OR Pelvic floor defects OR Anterior wall prolapse OR Posterior wall prolapse OR Enterocele OR Rectocele OR Uterine prolapse OR Cystocele OR Procidentia OR Pelvic organ OR Pelvic organ prolapse OR Gynecologic surgery OR hysterectomy OR Abdominal hysterectomy OR Vaginal hysterectomy OR Robotic hysterectomy OR Myomectomy OR Abdominal Myomectomy OR Vaginal Myomectomy OR Robotic Myomectomy OR Gynecologic laparoscopy OR Diagnostic gynecologic laparoscopy OR Operative gynecologic laparoscopy OR Diagnostic laparoscopy in gynecology OR Operative laparoscopy in gynecology OR Laparoscopy in gynecology OR Gynecologic laparotomy OR Laparotomy in gynecology OR Operative hysteroscopy OR Ovary OR Uterus OR Fallopian tubes OR Uterine tube OR Disorders menstruation OR Menstrual irregularities OR Irregularities menstruation OR Abnormal uterine bleeding OR Dysfunctional uterine bleeding OR Amenorrhea OR Dysmenorrhea OR Menorrhagia OR Menometrorrhagia OR Intermenstrual bleeding OR AUB OR DUG OR PALM-COEIN OR Postmenopausal bleeding OR Adenomyosis OR Surgery adenomyosis OR Ectopic pregnancy OR Tubal pregnancy OR Pregnancy unknown location OR PUL OR Pelvic inflammatory disease OR PID OR Cervicitis OR Tuboovarian abscess OR TOA OR Rectovaginal fistula OR Urinary tract fistula OR

© 2019 Brandt JS et al. *JAMA Network Open.*

Gynecologic fistula OR fistula OR Gestational trophoblastic disease OR Gestational trophoblastic neoplasia OR GTN OR GTD OR Molar pregnancy OR Hydatidiform mole OR Partial mole OR Complete mole OR Abortion OR Miscarriage OR Spontaneous abortion OR Incomplete abortion OR Complete abortion OR Septic abortion OR Spontaneous miscarriage OR Incomplete miscarriage OR Complete miscarriage OR Septic miscarriage OR SAB OR Medical management abortion OR Medical management first trimester loss OR Surgical management abortion OR Surgical management second trimester loss OR Inevitable abortion OR First trimester loss OR First trimester miscarriage OR First trimester abortion OR Early miscarriage OR Early abortion OR Threatened abortion OR Threatened miscarriage OR Dilation "AND" curettage OR D&C OR Dilation "AND" evacuation OR D&E OR OB GYN OR Obstetric OR Obstetrics OR Preconception OR Preconception care OR Preconception counseling OR Preconception genetic counseling OR obstetrics "AND" gynecology OR Teratogens OR Teratogenesis OR Organogenesis OR Congenital malformations OR Birth defect OR Congenital anomaly OR Hyperemesis OR Nausea "AND" vomiting pregnancy OR Hyperemesis gravidarum OR HEG OR 2nd trimester loss OR 3rd trimester loss OR Second trimester loss OR Third trimester loss OR 2nd trimester abortion OR Second trimester abortion OR Intrauterine fetal demise OR 2nd tri abortion OR Second tri abortion OR Stillbirth OR Fetal demise OR Fetal loss OR Fetus OR Fetal OR Neonate OR Fetal development OR development fetus OR Fetal malformation OR Multifetal gestation OR Multifetal pregnancy OR Multiple gestation OR Multiple pregnancy OR Twin gestation OR Higher order multiple OR Amniocentesis OR Invasive genetic testing in pregnancy OR Prenatal diagnosis OR Chorionic villus sampling OR CVS OR Cell free DNA OR Cell free fetal DNA OR NIPT OR NIPS OR First trimester genetic screening OR First trimester genetic testing OR First trimester screen OR Aneuploidy screening OR Second trimester screen OR Quad screen OR Quadruple screen OR Triple screen OR Maternal serum acylate OR Aneuploidy screening OR Down syndrome screening OR Obstetric ultrasound OR Ultrasound in pregnancy OR Ultrasound in obstetrics OR Obstetric ultrasonography OR Ultrasonography in obstetrics OR Preterm labor OR PTL OR Premature rupture membranes OR PROM OR Premature rupture membranes at term OR Term premature rupture membranes OR Term PROM OR Preterm premature rupture membranes OR PPROM OR Preterm PROM OR Cerclage OR History indicated cerclage OR Ultrasound indicated cerclage OR Emergency cerclage OR Rescue cerclage OR Cervical insufficiency OR Cervical incompetence OR Short cervix OR Cervical shortening OR Preterm delivery OR PTD OR Previable delivery OR Preterm birth OR Late preterm delivery OR Post term pregnancy OR Post dates pregnancy OR Post term delivery OR Post-term OR Post-dates OR Postterm OR Postdates OR Induction labor OR IOL OR Labor induction OR Augmentation labor OR Labor augmentation OR Labor abnormalities Abnormalities laborer Labor dystocia OR Arrest dilation OR Arrest descent OR Protraction labor OR Protracted second stage labor OR Protracted first stage labor OR Protracted labor OR Failed induction OR Cephalopelvic disproportion OR CPD OR First stage arrest OR Second stage arrest OR Fetal heart rate abnormalities OR Nonreassuring fetal heart tracing OR Non-reassuring fetal heart tracing OR nrfht OR Fetal decelerations OR variable decelerations OR Late decelerations OR Minimal variability OR Absent variability OR Category one tracing OR Category two tracing OR Category three tracing OR Poor variability OR Fetal tachycardia OR Fetal bradycardia OR Fetal monitoring OR Electronic fetal monitoring OR Continuous fetal monitoring OR Breech presentation OR Face presentation OR Malpresentation in labor OR Malpresentation in pregnancy OR Fetal malpresentation OR Malpresentation OR Prolapsed cord OR Cord prolapse OR Umbilical cord entanglement OR Umbilical cord OR Operative vaginal deliveries OR Vacuum assisted vaginal delivery OR Forceps assisted vaginal delivery OR Obstetrical forceps OR Assisted second stage OR Obstetrical hemorrhage OR antepartum hemorrhage OR Intrapartum hemorrhage OR Postpartum hemorrhage OR Management postpartum hemorrhage OR Maternal OR Maternal morbidity OR Vaginal hematoma OR Perineal hematoma OR Maternal mortality OR Primary cesarean delivery OR Primary cesarean section OR Cesarean delivery OR Cesarean section OR Repeat cesarean delivery OR Repeat cesarean section OR Complications cesarean delivery OR Complications cesarean section OR Postoperative complications cesarean delivery OR Postoperative complications cesarean section OR Wound infection cesarean delivery OR Wound infection cesarean section OR Hematoma cesarean delivery OR Hematoma cesarean section OR Hemorrhage

© 2019 Brandt JS et al. *JAMA Network Open.*

cesarean section OR Hemorrhage cesarean delivery OR Puerperal infection OR Post cesarean endometritis OR Endometritis OR Post vaginal endometritis OR Post partum infection OR Postpartum infection OR Pelvic thrombophlebitis OR Septic pelvic thrombophlebitis OR Obstetric anesthesia OR Obstetric anesthesia OR OB anesthesia OR Complications OB anesthesia OR Complications anesthesia in obstetrics OR Post epidural hypotension OR Hypotension epidural OR Fetal heart rate abnormalities epidural OR Fetal heart tracing epidural OR Intrapartum infection OR Intra-amniotic infection OR Amnionitis OR Chorioamnionitis OR chorio OR Hypertensive disorders pregnancy OR Hypertension in pregnancy OR Preeclampsia OR PEC OR Pregnancy induced hypertension OR Gestational hypertension OR Chronic hypertension in pregnancy OR chen OR Chronic hypertension with superimposed preeclampsia OR Eclampsia OR Toxemia OR Toxemia pregnancy OR Pre-eclampsia OR Cardiovascular disease in pregnancy OR Pulmonary disease in pregnancy OR Renal disease in pregnancy OR Neurological disease in pregnancy OR Hematologic disease in pregnancy OR Endocrine disease in pregnancy OR Autoimmune disorders pregnancy OR Cardiovascular disease complicating pregnancy OR Pulmonary disease complicating pregnancy OR Renal disease complicating pregnancy OR Neurological disease complicating pregnancy OR Hematologic disease complicating pregnancy OR Endocrine disease complicating pregnancy OR Autoimmune disorders pregnancy OR Autoimmune disorders complicating pregnancy OR Infectious diseases in pregnancy OR Congenital infection OR Vertical transmission OR Zika virus OR Zika infection in pregnancy OR Zika OR Zika in pregnancy OR Group A streptococcus in pregnancy OR Varicella in pregnancy OR Pyelonephritis in pregnancy OR CMV in pregnancy OR Cytomegalovirus OR Toxoplasmosis in pregnancy OR Parvovirus in pregnancy OR Rubella in pregnancy OR Herpes in pregnancy OR TOR CH infection in pregnancy OR Pregnancies complicated by human immunodeficiency virus infection OR HIV in pregnancy OR HIV infection in pregnancy OR Human immunodeficiency virus infection in pregnancy OR Human immunodeficiency virus in pregnancy OR Pregnancy complicated by HIV OR Pregnancy complicated by HIV infection OR Pregnancy complicated by human immunodeficiency virus OR Pregnancy complicated by human immunodeficiency virus infection OR Pregnancy complicated by AIDS OR Acquired immunodeficiency syndrome in pregnancy OR Pregnancy complicated by acquired immunodeficiency syndrome OR Postpartum psychosis OR Post partum psychosis OR Post-partum psychosis OR Post-partum depression OR Post partum depression OR Psychiatric disease in pregnancy OR Baby blues OR Edinburgh OR Postpartum blues OR Post-partum blues OR Post partum blues OR Postpartum depression OR Abnormal fetal growth OR IUGR OR Intrauterine growth restriction OR Intrauterine growth retardation OR Fetal growth restriction OR FGR OR Macrosomia OR Accelerated fetal growth OR Restricted fetal growth OR Retarded fetal growth OR Fetal anomalies OR Fetal abnormalities OR Fetal malformation OR Congenital fetal anomalies OR Congenital fetal abnormalities OR Congenital fetal malformations OR Placental abnormalities OR Abnormalities placenta OR Placenta OR Trauma in pregnancy OR Diabetes in pregnancy OR Gestational diabetes OR Gestational diabetes mellitus OR Diabetes mellitus in pregnancy OR Shoulder dystocia OR Management shoulder dystocia OR Early term delivery OR Isoimmunization OR Maternal alloimmunization OR Maternal isoimmunization OR Rh isoimmunization OR Rh-isoimmunization OR Rh sensitization OR Hemolytic disease newborn OR Hemolytic disease fetus "AND" newborn OR Rh incompatibility OR Alloimmunization OR Isoimmunization in pregnancy OR Alloimmunization in pregnancy OR HDFN OR Uterine hyperplasia OR Hyperplasia uterus OR Endometrial hyperplasia OR Hyperplasia endometrium OR uterine malignancy OR Uterine cancer OR endometrial cancer OR endometrial malignancy OR ovarian cancer OR Ovarian malignancy OR Cancer ovary OR endometrial carcinoma OR uterine carcinoma OR ovarian carcinoma OR gynecologic malignancy OR gynecologic cancer OR gynecological cancer OR cancer in gynecology OR urinary incontinence OR urinary continence OR Parturition OR Menopausal OR Pelvic Floor OR Childbirth OR child birth OR menses OR menstrual OR Down Syndrome OR Downs Syndrome OR Downs-Syndrome OR Down-Syndrome OR FIGO OR FIGO staging OR (International Federation Gynecology AND Obstetrics OR Trisomy OR Ovarian failure OR IVF OR in-vitro fertilization OR in vitro fertilization OR invitro fertilization OR fertilization OR in vitro OR in-vitro OR In-vitro-

© 2019 Brandt JS et al. *JAMA Network Open.*

fertilization OR Embryo OR fertility OR assisted reproductive technology OR urogynecology OR urogyn OR progesterone OR estrogen OR uterine OR intrauterine OR ovarian OR reproduction))

© 2019 Brandt JS et al. *JAMA Network Open.*

eAppendix 3:

Search query by OBGYN journals in the ISI Web of Knowledge

SO=(Acta Obstetricia Et Gynecologica Scandinavica OR Adolescent "And" Pediatric Gynecology OR African Journal of Reproductive Health OR American Journal Of Obstetrics "And" Gynecology OR American Journal Of Perinatology American Journal of Reproductive Immunology OR Annales Chirurgiae Et Gynaecologiae Or Archives Of Gynecology "And" Obstetrics OR Australian "And" New Zealand Journal of Obstetrics & Gynaecology OR Baillieres Clinical Obstetrics "And" Gynaecology OR Best Practice "And" Research Clinical Obstetrics Gynaecology Or Birth-Issues In Perinatal Care Or Breast Or Bjog An International Journal Of Obstetrics "And" Gynaecology OR Breast Feeding Medicine OR British Journal Of Obstetrics "And" Gynaecology OR Climacteric OR Clinical Obstetrics "And" Gynecology OR Clinics In Perinatology OR Contemporary OB/GYN OR Contraception OR Contraception Fertilite Sexualite OR Gynecologic Oncology OR Current Obstetrics "And" Gynecology Reports OR Current Opinion In Obstetrics "And" Gynecology OR Current Problems In Obstetrics Gynecology "And" Fertility OR Early Human Development OR European Journal of Contraception "And" Reproductive Health Care OR European Journal Of Obstetrics Gynecology "And" Reproductive Biology OR European Journal of Gynaecological Oncology OR Female Pelvic Medicine "And" Reconstructive Surgery OR Fertility "And" Sterility OR Fetal Diagnosis "And" Therapy OR Geburtshilfe and Frauenheilkunde OR Gineco ro OR Ginecologia y Obstetricia Clinica OR Ginekologia Polska OR Gynakologe Or Gynakologisch-Geburtshilfliche Rundschau OR Gynecologic "And" Obstetric Investigation OR Gynecological Endocrinology OR Gynecologie Obstetrique Fertilite et Senologie OR Human Reproduction Update OR Human Reproduction OR Hypertension in Pregnancy OR International Breastfeeding Journal OR International Journal of Fertility "And" Sterility OR International Journal of Gynecological Cancer OR International Journal of Obstetric Anesthesia OR International Urogynecology Journal "And" pelvic Floor dysfunction OR International Journal Of Gynecology "And" Obstetrics OR International Journal of Gynecological Pathology OR International Journal of Womens Health "And" Reproduction Sciences OR Journal of Assisted Reproduction "And" Genetics OR JOURNAL OF THE American Association Of Gynecologic Laparoscopists OR Journal Of Gynecology Obstetrics "And" Human Reproduction OR Journal of Gynecologic Surgery OR Journal of human lactation OR Journal of Lower Genital Tract Disease OR Journal of Maternal-Fetal "And" Neonatal Medicine OR Journal Of Maternal-Fetal Investigation OR Journal of Midwifery "And" Womens health OR Journal Of Minimally Invasive Gynecology OR Journal Of Obstetrics "And" Gynaecology Research OR Journal of Ovarian Research OR Journal of Pediatric "And" Adolescent Gynecology OR Journal Of Perinatology OR Journal of Perinatal Medicine OR Journal Of Psychosomatic Obstetrics "And" Gynecology OR Journal of Reproductive Medicine OR Journal of Reproductive "And" infant psychology OR Journal Of The Society For Gynecologic Investigation OR Maternal "And" Child Nutrition OR Maternal "And" Child Health Journal OR Maturitas OR Menopause OR Molecular Human Reproduction OR Iranian Journal of Fertility "And" Sterility OR Midwifery OR Obstetrics "And" Gynecology Clinics Of North America OR Obstetrics "And" Gynecology OR Obstetrical "And" Gynecological Survey OR Perspectives on Sexual "And" Reproductive Health OR Placenta OR Paediatric "AND" Perinatal Epidemiology OR Prenatal Diagnosis OR Reproduction OR Reproductive Health OR Reproductive health matters OR Reproductive Sciences OR Revue de Médecine Périnatale OR SAJOG South African Journal Of Obstetrics "And" Gynaecology OR Seminars In Perinatology OR Seminars in Reproductive Medicine OR Sexual "And" reproductive healthcare OR Taiwanese Journal Of Obstetrics Gynecology OR Twin research "And" human genetics OR Ultrasound In Obstetrics "And" Gynecology OR Wideochirurgia I Inne Techniki Małoinwazyjne OR Women "And" Birth OR Zeitschrift fur Geburtshilfe und Neonatologie)

© 2019 Brandt JS et al. *JAMA Network Open.*

eTable 1:

100 top-cited articles from all journals

*Citations	First Author	Second Author	Senior Author	Title	Year	Journal
9204	Rossouw, JE	Anderson, GL	Ockene, J	Risks and benefits of estrogen plus progestin in healthy postmenopausal wom en - principal results from the Women's Health Initiative randomized controlled trial	2002	Journal of the American Medical Association
5631	Slamon, DJ	Godolphin, W	Press, MF	Studies of the her-2/neu proto-oncogene in human breast and ovarian cancer	1989	Science
5056	Walboomers, JM	Jacobs, MV	Munoz, N	Human papillomavirus is a necessary cause of invasive cervical cancer worldwide	1999	Journal of Pathology
4752	Cox, JL	Holden, JM	Sagovsky, R	Detection of postnatal depression - development of the 10-item Edinburgh postnatal depression scale	1987	British Journal of Psychiatry
4392	Hulley, S	Grady, D;	Vittinghoff, E	Randomized trial of estrogen plus progestin for secondary prevention of coronary heart disease in postmenopausal women	1998	Journal of the American Medical Association
4269	Miki, Y	Swensen, J	Skolnick, MH	A strong candidate for the breast and ovarian-cancer susceptibility gene BRCA 1	1994	Science
3871	Abrams, P	Cardozo, L	Wein, A	The standardisation of terminology of lower urinary tract function: report from the standardisation sub-Committee of the International Continence Society	2002	Neurology and Urodynamics
3568	Munoz, N	Bosch, FX	Meijer, CJLM	Epidemiologic classification of human papillomavirus types associated with cervical cancer	2003	New England Journal of Medicine
2921	Anderson, GL	Limacher, M	Wassertheil-Smoller, S	Effects of conjugated, equine estrogen in postmenopausal wome n with hysterectomy - the Women's Health Initiative randomized controlled trial	2004	Journal of the American Medical Association
2892	Curiel, TJ	Coukos, G	Zou, WP	Specific recruitment of regulatory T cells in ovarian carcinoma fosters immune privilege and predicts reduced survival	2004	Nature

© 2019 Brandt JS et al. *JAMA Network Open.*

2752	Goldenberg RL	Culhane, Jennifer F	Romero, Roberto	Epidemiology and causes of preterm birth	2008	Lancet
2616	Connor, EM	Sperling, RS	Balsley, J	Reduction of maternal-infant transmission of human-immunodeficiency-virus type-1 with zidovudine treatment	1994	New England Journal of Medicine
2599	Black, DM	Cummings, SR	Ensrud, KE	Randomised trial of effect of alendronate on risk of fracture in women with existing vertebral fractures	1996	Lancet
2587	Neer, RM	Arnaud, CD	Mitlak, BH	Effect of parathyroid hormone (1-34) on fractures and bone mineral density in postmenopausal women with osteoporosis.	2001	New England Journal of Medicine
2548	Bell, D	Berchuck, A	Thomson, E	Integrated genomic analyses of ovarian carcinoma	2011	Nature
2448	Bosch, FX	Manos, Mm	Wheeler, C	Prevalence of human papillomavirus in ce rvical cancer - a worldwide perspective	1995	Journal of the National Cancer Institute
2446	Bump, RC	Mattiasson, A	Smith, ARB	The standardization of terminology of fe male pelvic organ prolapse and pelvic flo or dysfunction	1996	American Journal of Obstetrics and Gynecology
2441	Rosen, R	Brown, C	D'Agostino, R	The female sexual function index (fsfi): a multidimensional self-report instrument for the assessment of female sexual function	2000	Journal of Sex and Marital Therapy
2271	Mcguire, WP	Hoskins, WJ	Davidson, M	Cyclophosphamide and cisplatin compared with paclitaxel and cisplatin in patients with Stage III and Stage IV ovarian cancer	1996	New England Journal of Medicine
2213	Petricoin, EF	Ardekani, AM	Liotta, LA	Use of proteomic patterns in serum to identify ovarian cancer	2002	Lancet
2175	Maynard, SE	Min, JY	Karumanchi, SA	Excess placental soluble fms-like tyrosine kinase 1 (sflt1) may contribute to endothelial dysfunction, hypertension, and proteinuria in preeclam psia	2003	Journal of Clinical Investigation

© 2019 Brandt JS et al. *JAMA Network Open.*

2166	Ettinger, B	Black, DM	Cummings, SR	Reduction of vertebral fracture risk in postmenopausal women with osteo porosis treated with raloxifene - results from a 3-year randomized clinical trial	1999	Journal of the American Medical Association
2163	Fauser, BCJM	Chang, J	Lobo, R	Revised 2003 consensus on diagnostic criteria and long-term health risks related to polycystic ovary syndrome (PCOS)	2004	Human Reproduction
2140	Chang, J	Azziz, R	Lobo, R	Revised 2003 consensus on diagnostic criteria and long-term health risks related to polycystic ovary syndrome	2004	Fertility and Sterility
2106	Wegmann, TG	Lin, H	Mosmann, TR	Bidirectional cytokine interactions in the maternal-fetal relationship - is successful pregnancy a th2 phenomenon	1993	Immunology Today
2087	Czeizel, AE	Dudas, I		Prevention of the 1st occurrence of neural-tube defects by periconceptional vitamin supplementation	1992	New England Journal of Medicine
2066	Palermo, G	Joris, H	Vansteirteghe m, AC	Pregnancies after intracytoplasmic injection of single spermatozoon into an oocyte	1992	Lancet
2001	Zur Hausen, H			Papillomaviruses and cancer: from basic studies to clinical application	2002	Nature Reviews Cancer
1946	Mendelsohn, ME	Karas, RH		The protective effects of estrogen on the cardiovascular s ystem	1999	New England Journal of Medicine
1937	Sibai, B	Dekker, G	Kupferminc, M	Pre-eclampsia	2005	Lancet
1936	Levine, RJ	Maynard, SE	Karumanchi, SA	Circulating angiogenic factors and the risk of preeclampsia	2004	New England Journal of Medicine
1922	Cummings, SR	Melton, LJ		Epidemiology and outcomes of osteoporo tic fractures	2002	Lancet

© 2019 Brandt JS et al. *JAMA Network Open.*

1913	Bosch, FX	Lorincz, A	Shah, KV	The causal relation between human papillomavirus and cervi cal cancer	2002	Journal of Clinical Pathology
1906	Antoniou, A	Pharoah, PDP	Easton, DF	Average risks of breast and ovarian cancer associated with brca1 or brca2 mutations detected in case series unselected for family history: a combined analysis of 22 studies	2003	American Journal of Human Genetics
1883	Grady, D	Rubin, SM	Cummings, SR	Hormone-therapy to prevent disease and prolong life in postmenopausal women	1992	Annals of Internal Medicine
1879	Metzger, BE	Lowe, LP	Sacks, DA	Hyperglycemia and adverse pregnancy ou tcomes	2008	New England Journal of Medicine
1867	Wald, N			Prevention of neural-tube defects - results of the Medical Research Council Vitamin Study	1991	Lancet
1859	Solomon, D	Davey, D	Young, N	The 2001 Bethesda system - terminology for reporting results of cervical cytology	2002	Journal of the American Medical Association
1859	Olsen, AL	Smith, VJ	Clark, AL	Epidemiology of surgically managed pelvic organ prolapse and urinar y incontinence	1997	Obstetrics and Gynecology
1856	Banks, E	Beral, V		Breast cancer and hormone-replacement therapy in the Million Women Study	2003	Lancet
1850	Wilson, WA	Gharavi, AE	Khamashta, MA	International consensus statement on preliminary classification criteria for definite antiphospholipid syndrome - report of an international workshop	1999	Arthritis and Rheumatism
1809	Stampfer, MJ	Colditz, GA	Hennekens, CH	Postmenopausal estrogen therapy and car diovascular-disease - 10-year follow-up from the nurses health study	1991	New England Journal of Medicine
1803	Pappas, PG	Kauffman, CA	Sobel, Jack D	Clinical practice guidelines for the management of candidiasis: 2009 update by the Infectious Diseases Society of America	2009	Clinical Infectious Diseases

© 2019 Brandt JS et al. *JAMA Network Open.*

1785	Barker, DJP			Fetal origins of coronary heart-disease	1995	British Medical Journal
1781	Bast, RC	Klug, TL	Knapp, RC	A radioimmunoassay using a monoclonal antibody to monitor the course of epithelial ovarian cancer	1983	New England Journal of Medicine
1777	Baker, J	Liu, JP		Role of insulin like growth factors in embryonic and postnatal growth	1993	Cell
1769	Mosselman, S	Polman, J	Dijkema, R	Er beta:<br>identification and characterization of a no vel human estrogen receptor	1996	FEBS Letters
1766	Hales, CN	Barker, DJP	Winter, PD	Fetal and infant growth and impaired glucose-tolerance at age 64	1991	British Medical Journal
1744	Ohara, MW	Swain, AM		Rates and risk of postpartum depression - a meta-analysis	1996	International Review of Psychiatry
1744	Durst, M	Gissmann, L		A papillomavirus DNA from a cervical-carcinoma and its<br>prevalence in cancer biopsy samples from different geographic regions	1983	Proceedings of the National Academy of Sciences of the United States of America-Biological Sciences
1731	Liberman, UA	Weiss, SR	Karpf, DB	Effect of oral alendronate on bone-mineral<br>density and the incidence of fractures inp ostmenopausal osteoporosis	1995	New England Journal of Medicine
1730	De Villiers, EM	Fauquet, C	Zur Hausen, H	Classification of papillomaviruses	2004	Virology
1706	Dunaif, A			Insulin<br>resistance and the polycystic ovary syndr ome:<br>mechanism and implications for pathogen esis	1997	Endocrine Reviews
1706	Klibanski, A	Adams-Campbell, L	Russell, WE	Osteoporosis<br>prevention, diagnosis, and therapy	2001	Journal of the American Medical Association

© 2019 Brandt JS et al. *JAMA Network Open.*

1676	Barker, DJP	Hales, CN	Clark, PMS	Type 2 (non-insulin-dependent) diabetes-mellitus, hypertension and hyperlipemia (syndrome-x) - relation to reduced fetal growth	1993	Diabetologia
1663	Nugent, R	Krohn, M	Hillier, Sl	Reliability of diagnosing bacterial vaginosis is improved by a standardized method of gram stain interpretation	1991	Journal of Clinical Microbiology
1656	Zhang, L	Conejo-Garcia, JR	Coukos, G	Intratumoral t cells, recurrence, and survival in epithelial ovarian cancer	2003	New England Journal of Medicine
1648	Harris, ST	Watts, NB	Miller, PD	Effects of risedronate treatment on vertebral and nonvertebral fractures in women with postmenopausal osteoporosis - a randomized controlled trial	1999	Journal of the American Medical Association
1647	Burge, R	Dawson, H	Tosteson, A	Incidence and economic burden of osteoporosis-related fractures in the united states, 2005-2025	2007	Journal of Bone and Mineral Research
1626	Ho, GY	Bierman, R	Burk, RD	Natural history of cervicovaginal papillomavirus infection in young women	1998	New England Journal of Medicine
1620	Gluckman, PD	Hanson, MA	Thornburg, KL	Effect of in utero and early-life conditions on adult health and disease	2008	New England Journal of Medicine
1589	Gluckman, PD	Godfrey, KM	Robinson, JS	Fetal nutrition and cardiovascular-disease in adult life	1993	Lancet
1586	Wakayama, T	Perry, ACF	Yanagimachi, R	Full-term development of mice from enucleated oocytes injected with cumulus cell nuclei	1998	Nature
1576	Khan, KS	Wojdyla, D		WHO analysis of causes of maternal death: a sys tematic review	2006	Lancet

1557	Cummings, SR	Black, DM		Effect of alendronate on risk of fracture in women with low bone density but without vertebral fractures - results from the fracture intervention trial	1998	Journal of the American Medical Association
1554	Metzger, BE	Gabbe, SG		International Association of Diabetes and Pregnancy St udy groups recommendations on the diagnosis and classification of hyperglycemia in pre gnancy	2010	Diabetes Care
1539	Black, DM	Delmas, P	Cummings, SR	Once-yearly zoledronic acid for treatment of postmenopausal oste oporosis	2007	New England Journal of Medicine
1536	Struewing, JP	Hartge, P	Tucker, MA	The risk of cancer associated with specifi c mutations of brca1 and brca2 among Ashkenazi Jews	1997	New England Journal of Medicine
1536	Rose, PG	Bundy, BN	Insalaco, S	Concurrent cisplatin-based radiotherapy and chemotherapy for locall y advanced cervical cancer	1999	New England Journal of Medicine
1530	Gimpl, G	Fahrenholz, F		The oxytocin receptor system: structure, function, and regulation	2001	Physiological Reviews
1525	Giudice, LC	Kao, LC		Endometriosis	2004	Lancet
1511	Tenter, AM	Heckeroth, AR	Weiss, LM	Toxoplasma gondii: from animals to humans	2000	International Journal for Parasitology
1510	Armstrong, DK	Bundy, B	Burger, RA	Intraperitoneal cisplatin and paclitaxel in ovarian cancer	2006	New England Journal of Medicine
1470	Stewart, CL	Kaspar, P	Abbondanzo, SJ	Blastocyst implantation depends on maternal expression of leukemia inhibitory factor	1992	Nature
1467	Crowther, CA	Hiller, JE	Robinson, JS	Effect of treatment of gestational diabetes mellitus on pregnancy outcomes	2005	New England Journal of Medicine
1461	Redman, CW	Sargent, IL		Latest advances in understanding preeclampsia	2005	Science

© 2019 Brandt JS et al. *JAMA Network Open.*

1456	Cormack, RS	Lehane, J		Difficult tracheal intubation in obstetrics	1984	Anaesthesia
1448	Alexander, GR	Himes, JHV	Kogan, M	A United States national reference for fetal growth	1996	Obstetrics and Gynecology
1447	Montoya, JG	Liesenfeld, O		Toxoplasmosis	2004	International Journal for Parasitology
1440	Abrams, P	Cardozo, L	Wein, A	The standardisation of terminology of lower urinary tract function: report from the standardisation sub-committee of the international continence society	2003	Urology
1431	Gifford, RW	August, PA	Taler, SJ	Report of the National High Blood Pressure Education Program Working Group on high blood pressure in pregnancy	2000	American Journal of Obstetrics and Gynecology
1427	Beral, V	Bull, D	Meirik, O	Breast cancer and hormone replacement therapy: collaborative reanalysis of data from 51 epidemiological studies of 52,705 women with breast cancer and 108,411 women without breast cancer	1997	Lancet
1423	Dawsonhughes, B	Harris, SS	Dallal, GE	Effect of calcium and vitamin d supplementation on bone, density in men and women 65 years of age or older	1997	New England Journal of Medicine
1409	Goldenberg, RL	Hauth, JC	Andrews, WW	Mechanisms of disease - intrauterine infection and preterm delivery	2000	New England Journal of Medicine
1409	Morris, M	Eifel, PJ	Mutch, DG	Pelvic radiation with concurrent chemotherapy compared with pelvic and para-aortic radiation for high-risk cervical cancer	1999	New England Journal of Medicine
1400	Creasman, WT	Morrow, CP	Heller, PB	Surgical pathological spread patterns of endometrial cancer- a gynecologic oncology group study	1987	Cancer

© 2019 Brandt JS et al. *JAMA Network Open.*

1395	Dominguez-Bello, MG	Costello, EK	Knight, R	Delivery mode shapes the acquisition and structure of the initial microbiota across multiple body habitats in newborns	2010	Proceedings of the National Academy of Sciences of the United States of America
1395	Fleming, DT	Wasserheit, JN		From epidemiological synergy to public health policy and practice: the contribution of ot her sexuallytransmitted diseases to sexual transmission of hiv infection	1999	Sexually Transmitted Infections
1388	Roberts, JM	Taylor, RN	Mclaughlin, Mk	Preeclampsia - an endothelial-cell disorder	1989	American Journal of Obstetrics and Gynecology
1378	Miller, V	Larosa, J	Kessler, G	Effects of estrogen or estrogen/progestin regimens on heart-disease risk-factors in postmenopausal women - the postmenopausal estrogen/progestin interventions (PEPI) trial	1995	Journal of the American Medical Association
1370	Ford, D	Easton, DF	Goldgar, D	Risks of cancer in BRCA 1 mutation carriers	1994	Lancet
1363	Rice, D	Barone, S		Critical periods of vulnerability for the developing nervous system: evidence from humans and animal models	2000	Environmental Health Sciences
1356	Hockel, M	Schlenger, K	Vaupel, P	Association between tumor hypoxia and malignant progression in advanced cancer of the uterine cervix	1996	Cancer Research
1354	Franks, S			Medical progress - polycystic-ovary-syndrome	1995	New England Journal of Medicine
1353	Cummings, SR	San Martin, J		Denosumab for prevention of fractures in postmenopausal women with osteoporosis	2009	New England Journal of Medicine
1353	Cummings, SR	Eckert, S	Jordan, VC	The effect of raloxifene on risk of breast cancer in postmenopausal women - results from the more randomized trial	1999	Journal of the American Medical Association

© 2019 Brandt JS et al. *JAMA Network Open.*

1344	Lubahn, DB	Moyer, JS	Smithies, O	Alteration of reproductive function but not prenatal sexual development after insertional disruption of the mouse estrogen-receptor gene	1993	Proceedings of the National Academy of Sciences of the United States of America
1343	Azziz, R	Woods, KS	Yildiz, BO	The prevalence and features of the polycy stic ovary syndrome in an unselected population	2004	Journal of Clinical Endocrinology and Metabolism
1343	Manson, JE	Hsia, J	Makela, P	Estrogen plus progestin and the risk of coronary heart di sease	2003	New England Journal of Medicine
1340	Manolagas, SC			Birth and death of bone cells: basic regulatory mechanisms and implications for the pathogenesis and treatment of osteoporosis	2000	Endocrine Reviews
*All articles are ranked according to the number of citations received in ISI Web of Knowledge.						

© 2019 Brandt JS et al. *JAMA Network Open.*

eTable 2:

Frequent journals of 100 top-cited articles from all journals (listed if ≥2 articles were in top-cited list)

Journal Name	Number of cited articles
New England Journal of Medicine	25
Lancet	14
Journal of the American Medical Association	10
Nature	5
Science	3
American Journal of Obstetrics and Gynecology	3

© 2019 Brandt JS et al. *JAMA Network Open.*

eTable 3:

Frequent authors of 100 top-cited articles from all journals (based on 1st, 2nd, and senior authorship and included if ≥2 papers in top-cited list)

Author	Number of cited articles
Cummings, SR	8
Black, DM	4
Barker, DJP	3
Bosch, FX	3
Cardozo, L	2
Anderson, GL	2
Azziz, R	2
Beral, V	2
Bundy, BN	2
Chang, J	2
Dawson Hughes, B	2
Abrams, P	2
Easton, DF	2
Gluckman, PD	2
Goldenberg, RL	2
Grady, D	2
Hales, CN	2
Karumanchi, SA	2
Lobo, R	2
Maynard, SE	2
Metzger, Boyd E	2
Munoz, N	2
Robinson, JS	2
Zur Hausen, H	2

© 2019 Brandt JS et al. *JAMA Network Open.*

eTable 4:

Institutions of origin of 100 top-cited articles from all journals (listed if ≥2 articles were in top-cited list)

Institution	Country	Number of cited articles
University of California-San Francisco	US	10
National Institutes of Health	US	8
Harvard University	US	4
University of Alabama	US	3
University of Southampton	UK	3

© 2019 Brandt JS et al. *JAMA Network Open.*

eTable 5:

100 top-cited articles from OBGYN journals

*Citations	First Author	Second Author	Senior Author	Title	Year	Journal
2446	Bump, RC	Mattiasson, A	Smith, ARB	The standardization of terminology of female p elvic organ prolapse and pelvic floor dysfunctio n	1996	American Journal of Obstetrics and Gynecology
2163	Fauser, BCJM	Chang, J	Lobo, R	Revised 2003 consensus on diagnostic criteria and long-term health risks related to polycystic ovary syndrome (PCOS)	2004	Human Reproduction
2140	Chang, J	Azziz, R	Lobo, R	Revised 2003 consensus on diagnostic criteria and long-term health risks related to polycystic ovary syndrome	2004	Fertility and Sterility
1859	Olsen, AL	Smith, VJ	Clark, AL	Epidemiology of surgically managed pelvic organ prolapse and urinary inc ontinence	1997	Obstetrics and Gynecology
1448	Alexander, GR	Himes, JHV	Kogan, M	A United States national reference for fetal growth	1996	Obstetrics and Gynecology
1431	Gifford, RW	August, PA	Taler, SJ	Report of the National High Blood Pressure Education Program Working Group on high blood pressure in pregnancy	2000	American Journal of Obstetrics and Gynecology
1395	Fleming, DT	Wasserheit, JN		From epidemiological synergy to public health policy and practice: the contribution of other se xuallytransmitted diseases to sexual transmissio n of hiv infection	1999	Sexually Transmitted Infections
1388	Roberts, JM	Taylor, RN	Mclaughl in, Mk	Preeclampsia - an endothelial-cell disorder	1989	American Journal of Obstetrics and Gynecology
1245	Skakkebae k, NE	Rajpert-De ME	Main, KM	Testicular dysgenesis syndrome: an increasingly common developmental disorder with environmental aspects	2001	Human Reproduction
1217	Gavin, NI	Gaynes, BN	Swinson, T	Perinatal depression - a systematic review of prevalence and incidence	2005	Obstetrics and Gynecology

© 2019 Brandt JS et al. *JAMA Network Open.*

1183	Pecorelli, S			Revised FIGO staging for carcinoma of the vulva, cervix, and endometrium	2009	International Journal of Gynecology and Obstetrics
1167	Finner, LB	Henshaw, SK		Disparities in rates of unintended pregnancy in the united states, 1994-2001	2006	Perspectives on Sexual and Reproductive Health
1166	Taylor, DD	Gercel-Taylor, C		Micro RNA signatures of tumor-derived exosomes as diagnostic biomarkers of ovarian cancer	2008	Gynecologic Oncology
1156	Roberts, JM	August, PA	Ngaiza, K	Hypertension in pregnancy report of the Ameri can College of Obstetricians and Gynecologists' task force on hypertension in pregnancy	2013	Obstetrics and Gynecology
1154	Khong, TY	Dewolf, FV	Brosens, I	Inadequate maternal vascular-response to placentation in pregnancies complicated by preeclampsia and by small-for-gestational-age infants	1986	British Journal of Obstetrics and Gynecology
1124	Hadlock, FP	Harrist, RB	Park, SK	Estimation of fetal weight with the use of head, body, and femur measurements – a prospective-study	1985	American Journal of Obstetrics and Gynecology
1058	Redman, CWG	Sacks, GP	Sargent, IL	Preeclampsia: an excessive maternal inflammatory response to pregnancy	1999	American Journal of Obstetrics and Gynecology
1055	Buttram, VC	Reiter, RC		Uterine leiomyomata-etiology, symptoms, and management	1981	Fertility and Sterility
1054	Carpenter, MW	Coustan, DR		Criteria for screening-tests for gestational diabetes	1982	American Journal of Obstetrics and Gynecology
1042	Canis, M	Donnez, JG		Revised American Society For Reproductive Medicine classification of endometriosis: 1966	1997	Fertility and Sterility
1023	Bokhman, JV			2 pathogenic types of endometrial carcinoma	1983	Gynecologic Oncology
1015	Davey, DA	Macgillivray, I		The classification and definition of the hypertensive disorders of pregnancy	1988	American Journal of Obstetrics and Gynecology

998	Morrow, CP	Bundy, BN	Graham, JE	Relationship between surgical pathological risk factors and outcomes in clinical Stage I and Stage II carcinoma of the endometrium- a Gynecologic Oncology Group study	1991	Gynecologic Oncology
984	Vansteirteghem, AC	Nagy, Z	Devroey, P	High fertilization and implantation rates after intracytoplasmic sperm injection	1993	Human Reproduction
973	Brown, MA	Lindheimer, MD	Moutquin, JM	The classification and diagnosis of the hypertensive disorders of pregnancy: statement from the International Society for the Study of Hypertension in Pregnancy (ISSHP)	2001	American Journal of Obstetrics and Gynecology
944	Weinstock, H	Berman, S	Cates W	Sexually transmitted diseases among American youth: incidence and prevalence estimates, 2000	2004	Perspective on Sexual and Reproductive Health
892	Kruger, TF	Menkveld, R	Smith, K	Sperm morphological features as a prognostic factor in in vitro fertilization	1986	Fertility and Sterility
873	Weinstein, L			Syndrome of hemolysis, elevated liver-enzymes, and low platelet count - a severe consequence of hypertension in pregnancy	1982	American Journal of Obstetrics and Gynecology
862	Keys, HM	Roberts, JA	Bell, JG	A phase III trial of surgery with or without adjunctive external pelvic radiation therapy in intermediate risk endometrial adenocarcinoma: a gynecologic oncology group study	2004	Gynecologic Oncology
852	Kruger, TF	Acosta, AA	Oehninger, S	Predictive value of abnormal sperm morphology in in vitro fertilization	1988	Fertility and Sterility
845	Duley, L			The global impact of pre-eclampsia and eclampsia	2009	Seminars in Perinatology
840	Östör, AG			Natural history of cervical intraepithelial neoplasia-a critical review	1993	International Journal of Gynecological Pathology
837	Benedet, JL	Bender, H	Pecorelli, S	FIGO staging classifications and clinical practice guidelines in the management of gynecologic cancers. FIGO Committee On Gynecologic Oncology.	2000	International Journal of Gynecology and Obstetrics

827	Lorincz, AT			Human papillomavirus infection of the cervix - relative risk associations of 15 common anogenital types	1992	Obstetrics and Gynecology
824	Golding, J	Pembrey, M	Jones, R	Alspac-the avon longitudinal study of parents and children-study methodology	2001	Paediatric and perinatal epidemiology
781	Kennedy, S	Bergqvist, A	Saridogan, E	ESHRE guideline for the diagnosis and treatment of endometriosis	2005	Human Reproduction
779	Bland, JM	Altman, DG		Applying the right statistics: analyses of measurement studies	2003	Ultrasound in Obstetrics and Gynecology
778	Manning, JT	Scutt, D	Lewis-Jones, DI	The ratio of 2nd to 4th digit length: a predictor of sperm numbers and concentrations of testosterone, lutenizing hormone and oestrogen	1998	Human Reproduction
776	Azziz, R	Carmina, E	Witchel, Seline F	The Androgen Excess and PCOS Society criteria for the polycystic ovary syndrome: the complete task force report	2009	Fertility and Sterility
773	Faddy, MJ	Gosden, RG	Nelson, JF	Accelerated disappearance of ovarian follicles in midlife-implications for forecasting menopause	1992	Human Reproduction
763	Cooper, TG	Noonan, E	Vogelsong, KM	World Health Organization reference values for human semen characteristics	2010	Human Reproduction
762	Eskenazi, B	Warner, ML		Epidemiology of endometriosis	1997	Obstetrics and Gynecology Clinics of North America
740	Bolvin, J	Bunting, L	Nygren, KG	International estimates of infertility prevalence and treatment seeking: potential need and demand for infertility medical care	2007	Human Reproduction
718	Bast, RC			The CA-125 tumor associated antigen-a review of the literature	1989	Human Reproduction
711	Bennett, HA	Einarson, A	Einarson, TR	Prevalence of depression during pregnancy: systematic review	2004	Obstetrics and Gynecology

© 2019 Brandt JS et al. *JAMA Network Open.*

703	Baird, DD	Dunson, DB	Schectman, JM	High cumulative incidence of uterine leiomyoma in black and white women: ultrasound evidence	2003	American Journal of Obstetrics and Gynecology
701	Williams, RL	Creasy, RK	Tashiro, M	Fetal growth and perinatal viability in California	1982	Obstetrics and Gynecology
696	Grady, D	Gebretsadik, T	Petitti, D	Hormone replacement therapy and endometrial cancer risk - a metaanalysis	1995	Obstetrics and Gynecology
688	Finer, LB	Zolna, MR		Unintended pregnancy in the United States: incidence and disparities, 2006	2011	Contraception
671	Evenson, DP	Jost, LK	Claussen, OP	Utility of sperm chromatin structure assay as a diagnostic and prognostic tool in the human fertility clinic	1999	Human Reproduction
671	Gomez, R	Romero, R	Berry, SM	The fetal inflammatory response syndrome	1998	American Journal of Obstetrics and Gynecology
670	Hesse, DG	Tracey, KJ	Lowry, SF	Cytokine appearance in human endotoxemia and primate bacteremia	1988	Surgery Gynecology and Obstetrics
669	Guerin, P	El Mouatassim, S	Menezo, Y	Oxidative stress and protection against reactive oxygen species in the pre-implantation embryo and its surroundings	2001	Human Reproduction Update
669	Cedergren, MI			Maternal morbid obesity and the risk of adverse pregnancy outcome	2004	Obstetrics and Gynecology
663	Coulam, C B	Adamson, S C	Annegers, JF	Incidence of premature ovarian failure	1986	Obstetrics and Gynecology
663	Argawal, A	Saleh, RA	Bedaiwy, MA	Role of reactive oxygen species in the pathophysiology of human reproduction	2003	Fertility and Sterility
655	Weenen, C	Lavven, JSE	Themmen, APN	Anti-mullerian hormone expression pattern in the human ovary: potential implications for initial and cycle follicle recruitment	2004	Molecular Human Reproduction
649	Bavister, BD			Culture of preimplantation embryos: facts and artifacts	1995	Human Reproduction Update

© 2019 Brandt JS et al. *JAMA Network Open.*

639	Jackson, RA	Gibson, KA	Croughan, MS	Perinatal outcomes in singletons following in vitro fertilization: a meta-analysis	2004	Obstetrics and Gynecology
636	Quinn, P	Kerin, JF		Improved pregnancy rate in human in vitro fertilization with the use of a medium based on the composition of human tubal fluid	1985	Fertility and Sterility
635	Aalders, J	Abeler, V	Onsrud, M	Postoperative external irradiation and prognostic parameters in Stage-I endometrial carcinoma - clinical and histopathologic study of 540 patients	1980	Obstetrics and Gynecology
635	Nisolle, M	Donnez, JG		Peritoneal endometriosis, ovarian endometriosis, and adenomyotic nodules of the rectovaginal septum are three different entities	1997	Fertility and Sterility
633	Kelleher, CJ	Cardozo, LD	Salvatore, S	A new questionnaire to assess the quality of life of urinary incontinent women	1997	British Journal of Obstetrics and Gynecology
625	Hatch, R	Rosenfield, Rl	Tredway, D	Hirsutism - implications, etiology, and management	1981	American Journal of Obstetrics and Gynecology
624	Broekmans, FJ	Kwee, J	Lambalk, CB	A systematic review of tests predicting ovarian reserve and IVF outcome	2006	Human Reproduction Update
622	Silver, RM	Landon, MB	Mercer, BM	Maternal morbidity associated with multiple re peat cesarean deliveries	2006	Obstetrics and Gynecology
620	Sharpe, RM	Mckinnell, C	Fisher, JS	Proliferation and functional maturation of Sertoli cells, and their relevance to disorders of testis function in adulthood	2003	Reproduction
618	Knight, P	Glister, C		TGF-beta superfamily members and ovarian follicle development	2006	Reproduction
614	Gibbs, RS	Romero, R	Sweet, RL	A review of premature birth and subclinical inf ection	1992	American Journal of Obstetrics and Gynecology

© 2019 Brandt JS et al. *JAMA Network Open.*

605	March, W	Moore, VM	Davies, MJ	The prevalence of polycystic ovary syndrome in a community sample assessed under contrasting diagnostic criteria	2010	Human Reproduction
604	Meekins, JW	Pijnenborg, R	Vanasshe, A	A study of placental bed spiral arteries and trophoblast invasion in normal and severe preeclamptic pregnancies	1994	British Journal of Obstetrics and Gynecology
602	Dicker, RC	Greenspan, JR	Ory, HW	Complications of abdominal and vaginal hyster ectomy among women of reproductive age in the united-states	1982	American Journal of Obstetrics and Gynecology
595	Delgado, G	Bundy, B	Major, F	Prospective surgical pathological study of disease free interval in patients with Stage Ib squamous cell carcinoma of the cervix- a gynecologic oncology group study	1990	Gynecologic Oncology
595	Van Rooj, IAJ	Broekmans, FJM	Themmen, APN	Serum anti-mullerian hormone levels: a novel measure of ovarian reserve	2002	Human Reproduction
590	Oates, M	Cantwell, R		Death from psychiatric causes	2011	British Journal of Obstetrics and Gynecology
590	Crowley, P	Chalmers, I		The effects of corticosteroid administration before preterm delivery - an overview of the evidence from controlled trials	1990	British Journal of Obstetrics and Gynecology
581	Munne, S	Alikani M	Cohen, J	Embryo morphology, developmental rates, and maternal age are correlated with chromosome abnormalities	1995	Fertility and Sterility
578	Gosden, RG	Baird, DT	Webb, R	Restoration of fertility to oophorectomized sheep by ovarian autografts stored at 196 degrees c	1994	Human Reproduction
577	Romero, R	Espinoza, J.		The preterm parturition syndrome	2006	British Journal of Obstetrics and Gynecology
577	Hendrix, SL	Clark, A		Pelvic organ prolapse in the women's health initiative: gravity and gravidity	2002	American Journal of Obstetrics and Gynecology

© 2019 Brandt JS et al. *JAMA Network Open.*

575	Trussel, J			Contraceptive failure in the United States	2011	Contraception
574	Giles, WB	Trudinger, BJ	Baird, PJ	Fetal umbilical artery flow velocity waveforms and placental resistance - pathological correlation	1985	British Journal of Obstetrics and Gynecology
572	Abrams, P	Cardozo, L	Wein, A	The standardisation of terminology of lower urinary tract function: report from the standardisation sub-committee of the international continence society	2002	American Journal of Obstetrics and Gynecology
572	Nijhuis, JG	Prehctl, HFR		Are there behavioral states in the human fetus	1982	Early Human Development
572	Halme, J	Hammond, MG	Talbert, LM	Retrograde menstruation in healthy women and in patients with endometriosis	1984	Obstetrics and Gynecology
569	Sibai, BM	Ramadan, MK	Friedman, SA	Maternal morbidity and mortality in 442 pregnancies with hemolysis, elevated liver-enzymes, and low platelets (HELLP-syndrome)	1993	American Journal of Obstetrics and Gynecology
568	Reich, H	Decaprio, J		Laparoscopic hysterectomy	1989	Journal of Gynecologic Surgery
567	Velde, ERT	Pearson, PL		The variability of female reproductive ageing	2002	Human Reproduction Update
563	Tracey, KJ	Lowry, SF	Shires, GT	Cachectin tumor-necrosis-factor induces lethal shock and stress hormone responses in the dog	1987	Surgery Gynecology and Obstetrics
560	Clandinin, MT	Chappell, JE	Chance, GW	Intrauterine fatty acid accretion rates in human brain implications for fatty acid requirements	1980	Early Human Development
559	Koninckx, PR	Meuleman, C	Cornillie, FJ	Suggestive evidence that pelvic endometriosis is a progressive disease, whereas deeply infiltrating endometriosis is associated with pelvic pain	1991	Fertility and Sterility
559	Heintz, APM	Odicino, F	Beller, U.	Carcinoma of the ovary	2006	International Journal of Gynecology and Obstetrics

559	Delancey, JOL			Structural support of the urethra as it relates to stress urinary-incontinence - the hammock hypothesis	1994	American Journal of Obstetrics and Gynecology
556	Crowley, PA			Antenatal corticosteroid-therapy - a metaanalysis of the randomized trials, 1972 to 1994	1995	American Journal of Obstetrics and Gynecology
550	Merkatz, IR	Nitowsky, HM	Johnson, WE	An association between low maternal serum alpha-fetoprotein and fetal chromosomal-abnormalities	1984	American Journal of Obstetrics and Gynecology
549	Eppig, JJ			Oocyte control of ovarian follicular development and function in mammals	2001	Reproduction
547	Makuuchi, M	Hasegawa, H	Yamazaki, S	Ultrasonically guided subsegmentectomy	1985	Surgery Gynecology and Obstetrics
546	Kutteh, WH			Antiphospholipid antibody-associated recurrent pregnancy loss: treatment with heparin and low-dose aspirin is superior to low-dose aspirin alone	1996	American Journal of Obstetrics and Gynecology
545	Higham, JM	Obrien, PMS	Shaw, RW	Assessment of menstrual blood-loss using a pictorial chart	1990	British Journal of Obstetrics and Gynecology
542	Wright, TC	Massad, S		2006 consensus guidelines for the management of women with abnormal cervical cancer screening tests	2007	American Journal of Obstetrics and Gynecology
*All articles are ranked according to the number of citations received in ISI Web of Knowledge.						

© 2019 Brandt JS et al. *JAMA Network Open.*

eTable 6:

Top-cited articles on both lists

*Citations	Top 100 rank	Top OBGYN rank	First Author	Second Author	Senior Author	Title	Year	Journal
2446	17	1	Bump, RC	Mattiasson, A	Smith, ARB	The standardization of terminology of female pelvic organ prolapse and pelvic floor dysfunction	1996	American Journal of Obstetrics and Gynecology
2163	23	2	Fauser, BCJM	Chang, J	Lobo, R	Revised 2003 consensus on diagnostic criteria and long-term health risks related to polycystic ovary syndrome (PCOS)	2004	Human Reproduction
2140	24	3	Chang, J	Azziz, R	Lobo, R	Revised 2003 consensus on diagnostic criteria and long-term health risks related to polycystic ovary syndrome	2004	Fertility and Sterility
1859	38	4	Olsen, AL	Smith, VJ	Clark, AL	Epidemiology of surgically managed pelvic organ prolapse and urinary incontinence	1997	Obstetrics and Gynecology
1448	78	5	Alexander, GR	Himes, JHV	Kogan, M	A United States national reference for fetal growth	1996	Obstetrics and Gynecology
1431	81	6	Gifford, RW	August, PA	Taler, SJ	Report of the national high blood pressure education program working group on high blood pressure in pregnancy	2000	American Journal of Obstetrics and Gynecology
1395	87	7	Fleming, DT	Wasserheit, JN		From epidemiological synergy to public health policy and practice: the contribution of other sexuallytransmitted diseases to sexual transmission of hiv infection	1999	Sexually Transmitted Infections
1388	89	8	Roberts, Jm	Taylor, Rn	Mclaughlin, Mk	Preeclampsia - an endothelial-cell disorder	1989	American Journal of Obstetrics and Gynecology

*All articles are ranked according to the number of citations received in ISI Web of Knowledge.

© 2019 Brandt JS et al. *JAMA Network Open.*

eTable 7:

Year-by-year distribution of the number of articles published in non-specialty journals and cited in the ISI Web of Knowledge's Science Citation Index Expanded from 1980-2018

Publication Year	No. of Cited Items	% cited items	No. articles in top-100	Publication Year	No. of Cited Items	% cited items	No. articles in top-100
1980	14264	0.378569	0	2000	95140	2.525031	6
1981	15148	0.40203	0	2001	96269	2.554995	3
1982	15959	0.423554	0	2002	99125	2.630793	7
1983	16409	0.435497	2	2003	104941	2.785151	7
1984	17132	0.454686	1	2004	112895	2.996251	9
1985	16720	0.443751	0	2005	119190	3.163322	3
1986	17855	0.473875	0	2006	126115	3.347112	2
1987	18489	0.490701	2	2007	134750	3.576286	2
1988	17323	0.459755	0	2008	144043	3.822924	3
1989	16714	0.443592	2	2009	150140	3.984739	2
1990	21720	0.576452	0	2010	159347	4.229095	2
1991	57644	1.529881	4	2011	167606	4.44829	1
1992	60292	1.600159	4	2012	176821	4.692857	0
1993	66180	1.756428	5	2013	186950	4.961683	0
1994	71763	1.904601	3	2014	191773	5.089686	0
1995	77301	2.051581	5	2015	198836	5.277139	0
1996	80990	2.149487	7	2016	208593	5.536091	0
1997	86296	2.29031	5	2017	213569	5.668155	0
1998	88554	2.350237	4	2018	212594	5.642278	0
1999	92425	2.452974	9				

© 2019 Brandt JS et al. *JAMA Network Open.*

eTable 8:

Year-by-year distribution of the number of articles published in OBGYN journals and cited in the ISI Web of Knowledge's Science Citation Index Expanded from 1980-2018

Publication Year	No. of Cited Items	% cited items	No. articles in top-100	Publication Year	No. of Cited Items	% cited items	No. articles in top-100
1980	1206	0.432497	2	2000	5997	2.150649	2
1981	1457	0.522511	2	2001	7014	2.515367	5
1982	1569	0.562676	5	2002	6656	2.386981	4
1983	1697	0.60858	1	2003	7662	2.747753	4
1984	1809	0.648745	2	2004	7914	2.838126	8
1985	1648	0.591007	4	2005	8638	3.097767	2
1986	1923	0.689628	3	2006	9885	3.544967	6
1987	2287	0.820166	1	2007	9902	3.551064	2
1988	2287	0.820166	3	2008	10556	3.785602	1
1989	2515	0.901932	3	2009	10209	3.661161	3
1990	2653	0.951421	3	2010	11360	4.073933	2
1991	4232	1.517684	2	2011	12914	4.63123	3
1992	4168	1.494732	3	2012	12447	4.463754	0
1993	4750	1.703449	3	2013	15015	5.384693	1
1994	4480	1.606622	3	2014	14490	5.196417	0
1995	5204	1.866263	4	2015	15560	5.580141	0
1996	5242	1.879891	3	2016	15449	5.540334	0
1997	6023	2.159974	5	2017	15779	5.658679	0
1998	6129	2.197987	2	2018	14234	5.10461	0
1999	5886	2.110843	3				

© 2019 Brandt JS et al. *JAMA Network Open.*

eTable 9:

Citations by publication year for articles published in non-specialty journals from 1980-2018

Publication Year	0	1-99	100-499	500-999	1000-9,999	>10,000	Total (n)
1980	4558	9224	460	22	0	0	14264
1981	4807	9886	444	6	5	0	15148
1982	5311	10222	408	16	2	0	15959
1983	5290	10631	474	7	6	1	16409
1984	5336	11265	513	12	6	0	17132
1985	5287	10903	504	22	4	0	16720
1986	5737	11531	561	18	8	0	17855
1987	5833	12016	614	20	6	0	18489
1988	4803	11862	629	25	4	0	17323
1989	3456	12581	651	18	8	0	16714
1990	4208	16496	972	37	7	0	21720
1991	6081	47033	4247	219	64	0	57644
1992	5144	50276	4603	196	73	0	60292
1993	6201	54340	5314	248	77	0	66180
1994	7052	58701	5639	294	77	0	71763
1995	8173	62528	6229	292	79	0	77301
1996	8927	65071	6555	351	86	0	80990
1997	10105	68361	7348	363	119	0	86296
1998	8661	71397	8008	367	121	0	88554
1999	9507	73982	8451	371	113	1	92425
2000	9146	76062	9383	423	126	0	95140
2001	9050	77494	9224	405	96	0	96269
2002	9760	79644	9196	400	125	0	99125
2003	10865	84367	9247	352	110	0	104941
2004	12691	90257	9490	349	108	0	112895
2005	13917	95465	9337	369	102	0	119190
2006	15477	101546	8679	316	97	0	126115
2007	16545	109816	8023	282	84	0	134750
2008	16992	119297	7399	275	80	0	144043
2009	17849	125403	6623	208	57	0	150140
2010	19747	133452	5941	169	38	0	159347
2011	20532	142342	4540	156	35	1	167606
2012	21748	151430	3512	101	30	0	176821
2013	24289	159873	2710	62	16	0	186950
2014	25181	164776	1768	40	8	0	191773
2015	29826	167935	1047	25	2	0	198835

© 2019 Brandt JS et al. *JAMA Network Open.*

2016	40011	168050	517	9	6	0	208593
2017	60650	152817	100	2	0	0	213569
2018	131173	81416	5	0	0	0	212594
**Totals**	629926 (16.7%)	2959748 (78.6%)	169365 (4.5%)	6847 (0.2%)	1985 (0.05%)	3 (0.00008%)	3767874

© 2019 Brandt JS et al. *JAMA Network Open.*

eTable 10:

Citations by publication year for articles published in OBGYN journals from 1980-2018

Publication Year	0	1-99	100-499	500-999	1000-9,999	>10,000	Total
1980	156	980	66	4	0	0	1206
1981	290	1102	63	1	1	0	1457
1982	282	1217	65	4	1	0	1569
1983	344	1291	62	0	0	0	1697
1984	287	1448	71	3	0	0	1809
1985	214	1342	87	4	1	0	1648
1986	295	1546	79	2	1	0	1923
1987	429	1777	81	0	0	0	2287
1988	311	1890	83	2	1	0	2287
1989	328	2097	87	2	1	0	2515
1990	335	2195	121	2	0	0	2653
1991	644	3409	178	1	0	0	4232
1992	388	3593	183	4	0	0	4168
1993	702	3846	199	3	0	0	4750
1994	432	3848	197	3	0	0	4480
1995	519	4428	253	4	0	0	5204
1996	585	4417	236	2	2	0	5242
1997	1280	4472	264	5	2	0	6023
1998	881	4968	276	4	0	0	6129
1999	884	4753	246	1	2	0	5886
2000	805	4880	308	3	1	0	5997
2001	1365	5327	317	3	2	0	7014
2002	963	5357	330	6	0	0	6656
2003	1649	5687	321	5	0	0	7662
2004	1701	5892	311	8	2	0	7914
2005	1879	6421	336	1	1	0	8638
2006	2745	6843	290	6	1	0	9885
2007	2682	6996	222	2	0	0	9902
2008	2599	7746	209	2	0	0	10556
2009	1942	8071	193	2	1	0	10209
2010	2858	8314	185	3	0	0	11360
2011	3476	9306	128	4	0	0	12914
2012	3666	8697	83	1	0	0	12447
2013	5964	8969	81	0	1	0	15015
2014	5105	9351	34	0	0	0	14490
2015	6438	9108	14	0	0	0	15560
2016	6999	8443	7	0	0	0	15449
2017	8953	6825	1	0	0	0	15779

© 2019 Brandt JS et al. *JAMA Network Open.*

2018	10882	3352	0	0	0	0	14234
**Total**	82257 (29.5%)	190204 (68.2%)	6267 (2.2%)	97 (0.03%)	21 (0.008%)	0	278846

© 2019 Brandt JS et al. *JAMA Network Open.*
